# Natural AChE Inhibitors from Plants and their Contribution to Alzheimer’s Disease Therapy

**DOI:** 10.2174/1570159X11311040004

**Published:** 2013-07

**Authors:** Ana Paula Murray, María Belén Faraoni, María Julia Castro, Natalia Paola Alza, Valeria Cavallaro

**Affiliations:** a INQUISUR-CONICET, Departamento de Química, Universidad Nacional del Sur, Bahía Blanca, Argentina;; b Research Member of CONICET;; c Research Member of CIC

**Keywords:** Alzheimer’s Disease, acetylcholinesterase inhibitors, secondary metabolites, plant extracts, essential oils.

## Abstract

As acetylcholinesterase (AChE) inhibitors are an important therapeutic strategy in Alzheimer’s disease, efforts are being made in search of new molecules with anti-AChE activity. The fact that naturally-occurring compounds from plants are considered to be a potential source of new inhibitors has led to the discovery of an important number of secondary metabolites and plant extracts with the ability of inhibiting the enzyme AChE, which, according to the cholinergic hypothesis, increases the levels of the neurotransmitter acetylcholine in the brain, thus improving cholinergic functions in patients with Alzheimer’s disease and alleviating the symptoms of this neurological disorder. This review summarizes a total of 128 studies which correspond to the most relevant research work published during 2006-2012 (1st semester) on plant-derived compounds, plant extracts and essential oils found to elicit AChE inhibition.

## INTRODUCTION

Alzheimer’s disease (AD) is a progressive neuro-degenerative disorder associated with memory impairment and cognitive deficit. It is characterized by low levels of acetylcholine in the brain of AD patients. According to the cholinergic hypothesis, the inhibition of acetylcholinesterase (AChE), an enzyme that catalyzes acetylcholine hydrolysis, increases the levels of acetylcholine in the brain, thus improving cholinergic functions in AD patients. Further-more, although the general consensus concludes that AChE inhibitors (AChEi) can alleviate AD symptoms, they neither delay nor reverse the disease progress. Most of the drugs currently available for the treatment of AD are AChEi: tacrine (**1**), donezepil (**2**), rivastigmine (**3**) and galanthamine (**4**), all of which have limited effectiveness and some kind of side effect [1]. Tacrine (**1**) and donepezil (**2**), both from synthetic origin, were the first drugs approved for the treatment of cognitive loss in AD patients by US-FDA in 1993 and 1996, respectively. Rivastigmine (**3**) was approved in 2000 (US-FDA) and was designed from the lead compound physostigmine, a natural AChEi alkaloid. Galanthamine (**4**), a natural alkaloid first obtained from *Galanthus* spp. was approved by US-FDA in 2001. Huperzine A (**5**), an alkaloid found in *Huperzia* spp., is an AChEi commercialized as a dietary supplement for memory support and it is used to treat AD symptoms in China. This alkaloid has been thoroughly studied with promising results yielded particularly from the evaluation of cognitive performance of animals as well as from studies on its efficacy, tolerance and safety.

Taking into account that inhibitors **3**, **4** and **5** are related to natural products and that AChEi are an important therapeutic strategy for the treatment of AD, many research groups have focused their studies on naturally-occurring compounds from plants as potential sources of either new or more effective AChEi. These studies led to the discovery of an important number of secondary metabolites as well as plant extracts, both of which are characterized by their ability to inhibit AChE. On the other hand, the fact that a significantly relevant number of research papers has been recorded in this field during the last decades can be clearly attributed to the development of colorimetric methods which allow a rapid and facile screening of a large number of samples. Ellman’s method is the most widely used for the detection of AChEi, even in complex mixtures, and for the quantification of anti-AChE inhibitory activity [2-6].

Several reviews on the newly discovered AChEi obtained from plants, fungus and marine organisms have also been published over the last years [7-10]. The majority of these AChEi belong to the alkaloid group, including indole, isoquinoline, quinolizidine, piperidine and steroidal alkaloids. On the other hand, several non-alkaloidal and potent AChEi have been obtained from natural sources, including terpenoids, flavonoids and other phenolic compounds. Interestingly, although literature demonstrates to be rich in the study on AChEi obtained from plants, this issue keeps on being the center of attention for research as confirmed by the increasing number of studies published every year. Therefore, the purpose of this review is to provide a comprehensive summary of the literature, particularly that published during 2006-2012 (1^st^ semester) on plant-derived compounds, plant extracts and essential oils which have been reported to inhibit AChE. Readers interested not only in previous findings but also in synthetic/semisynthetic AChEi or natural AChEi of fungal, marine or microbial origin are recommended to see the above-mentioned reviews i.e. [7-10]. For the sake of brevity and in order to focus our attention on the most relevant findings, only those research papers reporting quantified results (IC_50 _and/or percentage of inhibition at a given concentration) were included. Extracts or essential oils with IC_50_ > 0.5 mg/ml were considered weakly active and were therefore not taken into account in the present review. With a few exceptions, only molecules with IC_50_ < 50 µM have been considered. Furthermore, unless otherwise stated, those results on AChE inhibition included in the present review refer to *in vitro* assays carried out with AChE from electric eel.

## ALKALOIDS WITH AChE INHIBITORY ACTIVITY

The quinoline alkaloids 3-hydroxy-2,2,6-trimethyl-3,4,5,6-tetrahydro-2*H*-pyrano[3,2-c] quinoline-5-one (**6**), ribalinine (**7**) and methyl isoplatydesmine (**8**) isolated from the aerial parts of *Skimmia laureola* (Rutaceae) were found to be linear mixed inhibitors of AChE with *K_i_* = 110.0, 30.0 and 30.0 µM, respectively [11]. These alkaloids were also observed to evidence butyrylcholinesterase (BChE) inhibition. 

On the other hand, of the several alkaloids that were isolated from the active extracts of *Esenbeckia leiocarpa* (Rutaceae), leptomerine (**9**) and kokusaginine (**10**) with IC_50_ values of 2.5 and 46 µM, respectively, were observed to elicit AChE inhibitory activity [12]. The isolation of skimmianine (**11**), a furoquinoline alkaloid with very low AChE inhibitory activity, was also reported by the same authors. This alkaloid was observed in another Rutaceae, *Zanthoxylum nitidum*, exhibiting a moderate AChE inhibitory activity (IC_50_ = 8.6 µg/ml) [13].

*Nelumbo nucifera* is a well-known medicinal plant belonging to the Nelumbonaceae family which was studied due to its therapeutic potential [14]. N-methylasimilobine (**12**), an aporphine alkaloid with an IC_50_ = 1.5 µg/ml which was found to be a non-competitive inhibitor, was recently isolated from this plant [15]. In a random screening, two extracts of *Beilschmiedia* species were observed to exhibit AChE inhibition and a phytochemical study of *B. alloiophylla* and *B. kunstleri* revealed the presence of several alkaloids with IC_50_ values ranging between 2.0 and 10.0 µM [16]. The most potent AChEi were found to be 2-hydroxy-9-methoxyaporphine (**13**), laurotetanine (**14**), liriodenine (**15**) and oreobeiline (**16**) (IC_50_ = 2.0-5.0 µM), with anti-AChE activity comparable to huperzine A (IC_50_ = 1.8 µM). A significant AChE inhibitory activity was also observed in secoboldine (**17**), boldine (**18**), isoboldine (**19**), asimilobine (**20**) and 3-methoxynordomesticine (**21**) (IC_50_ = 8.4 - 10.0 µM).

Research on plants from the genus *Corydalis* (Papaveraceae) which are used for the treatment of memory dysfunction in folk medicine reported the presence of benzylisoquinoline alkaloids with anti-AChE activity [7]. The ethanolic extract obtained from the tuber of *C. turtschaninovii* previously found to elicit AChE inhibition was selected to carry out a chemical study which led to the isolation of the isoquinoline alkaloids stylopine (**22**), epiberberine (**23**), pseudodehydrocorydaline (**24**), pseudocopsitine (**25**) and pseudoberberine (**26**). In the assay with mouse brain cortex as a source of AChE enzyme, the IC_50_ values obtained for each of these alkaloids were 15.8, 6.5, 8.4, 4.3 and 4.5 µM, respectively [17]. In addition, alkaloids **25 **and **26**, the two most active compounds, were found to elicit anti-amnesic activity [17, 18]. Alkaloids with benzylisoquinoline skeleton from *Corydalis* species having aromatic methylenedioxy groups and a quaternary atom of nitrogen were observed to show the strongest AChE inhibition [7, 17, 18]. In a more recent work, six protoberberine alkaloids **23**, **27** - **31**, were identified in rhizomes of *Coptis chinensis* which are traditionally used in Chinese medicine for the treatment of various diseases. Coptidis rhizomes and their alkaloids were reported to have cognitive-enhancing and neuroprotective effects and the analysis of the anti-AChE activity of these alkaloids showed that the IC_50_ values of berberine (**27**), palmatine (**28**), jateorrhizine (**29**), coptisine (**30**) and groenlandicine (**31**) ranged between 0.44 and 0.80 µM while that of epiberberine (**23**) was slightly higher (IC_50_ = 1.07 µM) [19]. Of these alkaloids, compounds **27**, **30** and **31** were observed to have an aromatic methylenedioxy group. In this study groenlandicine (**31**) and berberine (**27**) were found to be the most active as BChE inhibitors and epiberberine (**23**) was observed to significantly inhibit β-secretase (BACE1) [19].

The alkaloids (+)**-**canadaline (**32**) and (+)**-**canadine (**33**), both isolated from *Corydalis cava *and with an IC_50 _= 20.1 and 12.4 µM, respectively*,* were observed to elicit a moderate inhibitory activity when tested with AChE from human blood [20].

On the other hand, *Stephania venosa* (Menispermaceae), a Thai medicinal plant, was found to show a high AChE inhibitory activity. The ethanolic extract of *S. venosa *was subjected to bioassay-guided fractionation to identify AChEi [21]. The following moderately active quaternary protoberberine alkaloids could be isolated: stepharanine (**34**), cyclanoline (**35**) and *N*-methyl stepholidine (**36**) with IC_50_ values of 14.10, 9.23 and 31.30 µM, respectively. A similar fractionation approach was followed to identify the compounds responsible for AChE inhibition in *Chelidonium majus *(Papaveraceae) [22]. Three active constituents were identified, namely 8-hydroxydihydrochelerythrine (**37**), 8-hydroxydihydrosanguinarine (**38**) and berberine (**27**). Compounds **37** and **38**, with no previous record as AChEi, were found to elicit significant anti-AChE activity with an IC_50_ = 0.61 and 1.37 µM, respectively.

Taspine (**39**) was isolated from the alkaloid-enriched extract obtained from *Magnolia x soulangiana*^ (Magnoliaceae) [23]^. This alkaloid was found not only to show a dose-dependent and long-lasting inhibitory effect on AChE (IC_50_ = 0.33 µM) but also to be more potent than galanthamine (IC_50_ = 3.2 µM) although its inhibitory activity is comparable to that of tacrine (IC_50_ = 0.22 µM). Similar observations were obtained when the *in vitro* assay was performed with human AChE (IC_50_ = 0.54 µM). Compound **39** resulted to be inactive against BChE, acting as a selective AChEi.

*Catharanthus roseus* (Apocynaceae) is a plant mainly known as a source of vincristine and vinblastine, two alkaloids found in its leaves and appreciated as anticancer compounds. Several other compounds with biological importance can be also found in *C. roseus. *For example, the alkaloid serpentine (**40**), isolated from the roots of this plant, was reported to be a potent *in vitro* AChEi (IC_50 _= 0.775 µM) compared with physostigmine (IC_50 _= 6.45 µM) [24]. 

A bioassay-guided fractionation from the stems of *Ervatamia hainanensis* (Apocynaceae), a plant used in traditional Chinese medicine, allowed the isolation of several monoterpenoid indole alkaloids, some of them showing a potent AChE inhibitory activity [25]. For example, coronaridine (**41**) and voacangine (**42**), differing from each other only by the methoxy group attached to the aromatic ring, were observed to have an IC_50_ = 8.6 and 4.4 µM, respectively, these values being similar to that of galanthamine (3.2 µM). On the other hand, 10-hydro-xycoronaridine (**43**) was found to evidence a reduced AChE inhibition (IC_50_ = 29 µM), which was attributed to the introduction of a hydroxyl group to the aromatic ring. The indole alkaloids coronaridine (**41**) and voacangine (**42**), both detected in the stalks of *Tabernaemontana australis *(Apocynaceae), had been formerly identified as AChEi but no inhibition values were reported [26].

The genus *Tabernaemontana *is known for the wide variety of unusual bioactive indole alkaloids it produces. Among them, the bisindole alkaloids isolated from *T. divaricata* roots are an interesting example of new structures with potent AChE inhibitory activity. The crude alkaloid extract obtained from the root of *T. divaricata* was found to yield four bisindole alkaloids **44** - **47** [27]. The analysis of AChE inhibition revealed that 19,20-dihydrotabernamine (**44**) and 19,20-dihydroervahanine A (**45**) strongly inhibit AChE, with an IC_50_ = 0.227 and 0.071 µM, respectively, thus showing that they are significantly more active than galanthamine (IC_50_ = 0.594 µM). The fact that inhibition was found to be higher for compound **45** than for compound **44** suggests that the introduction of a carbomethoxy group at C16’ increases the enzymatic inhibition. In addition, taking into account that conodurine (**46**) and tabernaelegantine (**47**) were found to show no activity in AChE, it was suggested that the substitution at C11’ and C12’ is relevant for AChE inhibitory activity [27].

*Uncaria rhynchophylla* (Rubiaceae) is a Chinese medicine herb used to treat epilepsy. The alkaloid fraction from *U. rhynchophylla *is known for its antiepileptic and neuroprotective effects. Geissoschizine methyl ether (**48**), a strong AChEi, as well as six other weakly active alkaloids were recently isolated from this herb [28]. The active compound **48** was observed to inhibit AChE in a reversible and non-competitive way with an IC_50_ = 3.7 µg/ml.

The study of AChE inhibitory activity of Brazilian apocynacea *Himatanthus lancifolius*, commonly known as “agoniada”, led to the identification of active extracts in this plant and allowed the isolation of uleine (**49**), an active indole alkaloid, at a high concentration in the alkaloid fraction. The IC_50_ value observed for this alkaloid was 0.45 µM [29].

As to the Amaryllidaceae family, phytochemical research conducted in the last decades on this family revealed several alkaloids with moderate or potent inhibition of AChE [3, 7, 30]. In the search of new natural sources of galanthamine and other Amaryllidaceae alkaloids with anti-AChE activity, bulbs and leaves of *Hippeastrum papilio* collected in the South of Brazil were studied. Galanthamine (**4**), the already known alkaloids narwedine (**50**), haemanthamine (**51**), 11-hydroxyvittatine (**52**), 8-*O*-demethylmaritidine (**53**) and vittatine (**54**) as well as the new alkaloid 11(-hydro-xygalanthamine (**55**) were all isolated and of all of them galanthamine was obtained in significant amounts [31]. Compound **55** was observed to elicit AChE inhibition as other galanthamine-type alkaloids do, with an IC_50 _= 14.5 µM. Furthermore, because habranthine, epimer of **55**, was observed to have an anti-AChE activity similar to that of galanthamine, it was concluded that β configuration at C11 is unfavorable for the interaction with AChE [3, 31]. Other potent AChEi, such as *N*-allylnorgalanthamine (**56**) and *N*-(14-methylallyl)norgalanthamine (**57**), were isolated from *Leucojum aestivum*, an amaryllidacea used for the industrial extraction of galanthamine [32]. *N*-alkylated galanthamine derivatives **56** and **57 **were isolated together with galanthamine (**4**), epinorgalanthamine (**58**), narwedine (**50**) and lycorine (**59**), from the mother liquors obtained after the industrial production of galanthamine. Alkaloids **56** and **57**, with IC_50 _values of 0.18 and 0.16 µM, respectively, resulted to be ten times more potent AChEi than galanthamine (IC_50 _= 1.82 µM).

The chemical investigation of *Galanthus rizehensis*, a wild-growing species from Turkey, allowed the isolation of two new Amaryllidaceae alkaloid *N*-oxides, incartine *N*-oxide (**60**) and lycorine *N*-oxide (**61**) and seven known alkaloids namely, 1-acetyl-β-carboline (**62**), incartine (**63**), *N*-trans feruloyltyramine (**64**), lycorine (**59**), *O*-methylnorbelladine (**65**), vittatine (**54**) and 11-hydroxyvittatine (**52**) [33]. The potential of these alkaloids as AChEi was analyzed but only incartine *N*-oxide (**60**) was observed to elicit a moderate inhibitory activity (IC_50_ = 34.50 µM), incartine (**63**) was observed to be weakly active (IC_50_ = 106.97 µM) and the other alkaloids were found to be inactive. In a bioassay-guided fractionation of an active extract obtained from bulbs of *Nerine bowdenii*, the Amaryllidaceae alkaloid undulatine (**66**) was identified as the most active component of the alkaloid fraction, with an IC_50_ = 37 µM [34].

Although benzylphenethylamine alkaloids were considered to belong exclusively to the Amaryllidaceae, some of them have been found to belong to other families [35]. A new example of this exception was found through the chemical investigation of *Hosta plantaginea* (Liliaceae) [36]. Seventeen benzylphenetylamine alkaloids, including five new alkaloids, **67**-**71**, along with twelve known compounds [7-deoxy-*trans*-dihydronarciclasine, *O*-methyllycorenine, albomaculine, haemanthamine, *O*-demethylhaemanthamine, 8-*O*-demethylmaritadine, haemanthidine, yemenine C, lycorine, pseudolycorine, ungeremine (**72**) and norsanguinine (**73**)] were obtained. Some of these alkaloids were analyzed to determine whether they are AChEi or not.. Ungeremine (**72**) (IC_50 _= 3.85 µM), norsanguinine (**73**) (IC_50 _= 1.43 µM) and 8-demethoxy-10-*O*-methylhostasine (**69**) (IC_50_ = 2.32 µM) were all found to be potent AChE inhibitors.

After the isolation of the potent AChEi huperzine A (**5**) from *Huperzia serrata* (Lycopodiaceae), several plants belonging to the genus *Lycopodium* have been investigated in an attempt to find alkaloids with unusual skeletons that could have AChE inhibitory activity [7, 8, 37].**Five new Lycopodium alkaloids, 11(-hydroxyfawcettidine (**74**), 2(,11(-dihydroxyfawcettidine (**75**), 8(,11(-dihydro-xyfawcettidine (**76**), 2β-hydroxylycothunine (**77**) and 8(-hydroxylycothunine (**78**), with the fawcettimine skeleton were isolated from *L. serratum, *along with three known alkaloids, lycothunine (**79**), serratine (**80**) and serratanidine (**81**) [38]. AChE inhibitory activity was analyzed for the alkaloid lycoposerramine-H (**82**) previously isolated from *L. serratum *[39] and for compounds **74**, **75**, **78**. Alkaloids **75 **and **82** were observed to inhibit AChE with an IC_50_ = 27.9 and 16.7 µM, respectively, while **74** and **78** were observed to show no anti-AChE activity. In another study, three new alkaloids (**83 **- **85**) were isolated from *L. carinatum*, a species collected in Malasya [40]. Carinatumins A (**83**) and B (**84**) were observed to inhibit AChE from bovine erythrocytes with an IC_50_ = 4.6 and 7.0 µM, respectively, whereas carinatumin C (**85**) was observed to show no inhibition (IC_50_ > 100 µM). Alkaloids **83** and **84 **were observed to exhibit an AChE inhibitory activity similar to that of huperzine A and huperzine B (IC_50_ = 0.8 and 8.0 µM). Alkaloids from *L. casuarinoides* were also isolated and three new compounds, lycoparins A-C (**86 **- **88**), were characterized, of which lycoparin C (**88**) was found to show a moderate AChE inhibitory activity (from bovine erythrocytes) with an IC_50_ = 25 µM [41]. Lycoparin A (**86**) and lycoparin B (**87**), both having a carboxylic acid at C-15 and one or two *N*-methyl groups, were found to show no inhibitory activity.

As to *Sarcococca* and *Buxus* species (Buxaceae), they are known to produce steroidal alkaloids, some of which were observed to evidence strong AChE inhibition [7, 42, 43]. New steroidal alkaloid AChEi from *S. saligna* and *S. hookeriana* were recently found. In the case of *S. saligna*, the study –which was a continuation of previous research [44, 45]– of the bioactive steroidal alkaloids of this species allowed the isolation of five new compounds (**89**-**93**) and two already known bases (**94 **and **95**) [46]. The new alkaloids 5,14-dehydro-*N*_a_-demethylsaracodine (**89**), 14-dehydro-*N*_a_-demethylsaracodine (**90**), 16-dehydrosarcorine (**91**), 2,3-dehydrosarsalignone (**92**) and 14,15-dehydro-sarcovagine D (**93**), as well as the known compounds sarcovagine C (**94**) and salignarine C (**95**) were analyzed as anti-AChE agents. Only **91**, **92** and **95** were observed to exhibit significant AChE inhibition (IC_50 _= 12.5, 7.0 and 19.7 µM, respectively). Compounds **89 **- **92**, **94** and **95** were also found to elicit strong and selective BChE inhibition [46]. The bioassay-guided chemical investigation of *S. hookeriana* allowed the isolation of two new pregnane-type steroidal alkaloids, hookerianamide H (**96**) and hookerianamide I (**97**) together with the known alkaloids *N*_a_-methylepipachysamine D (**98**), sarcovagine C (**94**) and dictyophlebine (**99**) [47]. Compounds **94**,** 96**,** 97**,** 98 **and **99 **were tested for their inhibitory properties towards AChE and all of them were observed to elicit significant inhibitory activity (IC_50_ 2.9 – 34.1 µM) as well as a potent anti-BChE activity (IC_50_ 0.3 – 3.6 µM). Further studies on *S. hookeriana* yielded two new 5α-pregnane-type steroidal alkaloids, hookerianamides J (**100**) and K (**101**) [48]. Furthermore, eight known steroidal alkaloids, hookerianamide H (**96**) and hookerianamide I (**97**), chonemorphine (**102**), *N*-methylpachysamine A (**103**), epipachysamine-*E*-5-en-4-one (**104**), vagenine A (**105**), 2,3-dehydrosarsalignone (**92**) and sarcovagine C (**94**), were isolated and characterized. Alkaloids **94**, **100**,** 101**,** 102**,** 103 **and **104 **were analyzed as AChEi. Compounds **100**, **101**, **102** and **103** were observed to inhibit AChE moderately (IC_50_ 22.1 – 48.5 µM) while **104** and **94** were found to be more active inhibitors (IC_50_ 9.9 and 8.1 µM, respectively).

Phytochemical research on *Buxus hyrcana* allowed the identification of several Buxus alkaloids with cholinesterase inhibitory activity [43, 49]. Three new triterpenoidal alkaloids, namely 17-oxo-3-benzoylbuxadine (**106**), buxhyrcamine (**107**) and 31-demethylcyclobuxoviridine (**108**) along with sixteen known compounds, all tested as AChEi, were isolated and characterized in a recent study on *B. hyrcana *collected from Iran [50]. Weak AChE inhibitory activity was observed for *N*_b_-dimethylcyclobuxoviricine (**109**), papillozine C (**110**), cyclobuxophylline O (**111**) and arbora-1,9(11)-dien-3-one (**112**) (IC_50_ = 35.4 - 47.9 µM). In the same *in vitro* assay, 17-oxo-3-benzoylbuxadine (**106**), buxhyrcamine (**107**), homomoenjodaramine (**113**), buxmicrophylline F (**114**), buxrugulosamine (**115**), moenjodaramine (**116**) and N_20_-formyl-buxaminol E (**117**) were observed to show moderate AChE inhibition (IC_50_ = 17.6 - 25.5 µM) while spirofornabuxine (**118**) was found to elicit a strong AChE inhibitory activity (IC_50_ = 6.3 µM).

The crude methanolic extract of *B. natalensis*, a plant used to improve memory in the elderly by traditional healers in South Africa, was found to elicit AChE inhibition (IC_50 _= 28 µg/mL).**The phytochemical study of this extract yielded seven compounds **119 **- **125 **which were found to show either moderate or strong AChE inhibition [51]. The alkaloids *O*^2^-natafuranamine (**119**), *O*^10^-natafuranamine (**120**), cyclonataminol (**121**) and 31-demethylbuxaminol (**122**) were isolated and characterized for the first time while buxaminol A (**123**) was isolated for the first time as a natural product. Buxafuranamide (**124**) and buxalongifolamidine (**125**) were already known compounds. Compounds **119**, **120 **and **124 **were observed to exhibit a significantly higher AChE inhibitory activity compared to the rest, with IC_50_ values of 3.0, 8.5, and 14.0 µM, respectively. Compounds **121**,** 122**, **123 **and **125 **were observed to be less effective as AChEi (IC_50 _= 22.9 – 30.2 µM).

The bulbs of *Fritillaria *species (Lilliaceae) which are known to be a traditional medicinal herb called “Beimu” in China are used as an antitussive, antiasthmatic and expectorant agent. In the past, in a chemical study carried out on alkaloids from *F. imperialis* bulbs new steroidal alkaloids with weak AChE inhibition and great selectivity towards BChE were identified [52]. Thus, taking into account this previous study, the bulbs from five *Fritillaria* species were studied and their alkaloids were identified and evaluated as cholinesterase inhibitors. Eighteen alkaloids were isolated and their effects on human whole blood cholinesterase were assayed. Results showed that *N*-demethyl-puqietinone (**126**) from *F. puqiensis*, hupeheninoside (**127**) from *F. hupehensis*, ebeiedinone (**128**) from *F. ebeiensis var. purpurea*, yibeinoside A (**129**) from *F. pallidiflora *and chuanbeinone (**130**) from *F. delavayi* showed good AChE inhibition, with IC_50_ values of 6.4, 16.9, 5.7, 6.5 and 7.7 µM, respectively. However, all of them were weaker AChEi than galanthamine (IC_50 _= 1.9 µM). Compounds **127**, **128**, **129 **and **130 **were found to be stronger inhibitors on plasma BChE than galanthamine, the positive control [53].

In addition, the following steroidal alkaloids: conessine (**131**), isoconessimine (**132**), conessimin (**133**), conarrhimin (**134**) and conimin (**135**) were isolated in a bioassay-guided fractionation from the seeds of *Holarrhena antidysenterica* (Apocynaceae), a common Tibetan drug [54]. Compounds **131**, **133**, **134 **and **135 **were identified as active constituents against AChE. Conessimin (**133**) was found to be the strongest AChE inhibitor with an IC_50 _= 4 µM whereas conessine (**131**), conarrhimin (**134**) and conimin (**135**) were found to be moderate AChE inhibitors (IC_50 _= 21 - 28 µM). These findings indicate that the elimination of the N-methyl group of pyrrolidine moiety induces a significant increase of activity while the cleavage of either one or two N-methyl groups at C-3 position reduces the inhibitory potency. Compound **133 **was selected for a kinetic study through which it was demonstrated that its AChE inhibitory activity is both reversible and non-competitive. Molecular docking simulations of these compounds with AChE helped to understand their interactions with AChE and were consistent with the experimental results obtained [54].

## NON-ALKALOIDAL COMPOUNDS WITH AChE INHIBITORY ACTIVITY

In spite of the fact that the majority of the most potent inhibitors known to date are alkaloids, several non-alkaloidal AChEi from the plant kingdom and with different structural characteristics (terpenoids, sterols, flavonoids and phenolic compounds, etc) have been recognized as promising lead compounds as anti-AD agents [7-10]. Until 2006 only a few diterpenoids demonstrated to inhibit AChE [7]. However, further recent research has reported a larger number of compounds belonging to this group with the ability to exert either moderate or strong AChE inhibitory activity. In addition, a new cassane diterpene named niloticane (**136**) was isolated from the ethyl acetate bark extract of *Acacia nilotica* subsp. *kraussiana *(Fabaceae), a plant used in African traditional medicine [55]. Niloticane (**136**) was found to show an AChE inhibitory activity similar to that of the positive control galanthamine (IC_50_ = 4 and 2 µM, respectively). In addition, one new (**137**) and six known (**138 **- **143**) labdane-type diterpenoids were identified as AChE inhibitors present in an active extract obtained from *Leonurus heterophyllus* (Lamiaceae) by bioassay-guided fractionation [56]. Anti-AChE activity in **137 **– **143 **was analyzed in rat brain cortex as a source of AChE enzyme. Leoheteronin A (**141**) and leopersin G (**143**), both having a 15,16 epoxy group, were observed to be strong inhibitors with IC_50_ values of 11.6 and 12.9 µM, respectively. The new compounds leoheteronin F (**137**) and leoheteronin D (**142**) were found to show moderate inhibition with IC_50_ values of 16.1 and 18.4 µM, respectively. Leoheterin (**138**), hispanone (**139**) and galeopsin (**140**), all having a furan ring at the side chain, were found to be weakly active (IC_50_ = 38.5 - 42.7 µM).

*Asparagus adscendens* (Asparagaceae) is a medicinal plant traditionally used as a nerve tonic and remedy for memory impairments in Pakistan. Conypododiol (**144**), which was isolated from the chloroform fraction of the methanolic extract of *A. adscendens*, was found to elicit AChE and BChE inhibition with an IC_50_ = 2.17 and 11.21 µM, respectively [57]. This dual cholinesterase inhibitor was also observed to show potential as a bivalent ligand in molecular docking studies. Four non-competitive AChEi **145 **– **148 **were obtained in the chemical investigation of *Ajuga bracteosa* (Lamiaceae), another medicinal plant from Pakistan [58]. The diterpenoid dihydroajugapitin (**148**) was found to be the most active against AChE with an IC_50_ = 14.0 µM. Compared to compound **148**, lupulin A (**147**), clerodinin A (**146**) and dihydroclerodin (**145**) were observed to be less efficient inhibitors (IC_50_ = 19.2, 26.5 and 35.2, respectively) and diterpenoids **145 **- **148** were observed to elicit BChE inhibition. These findings indicate that the presence of a methoxy group at C-15 increases cholinesterase inhibitory potential.

From the methanolic extract of *Haloxylon recurvum* (Chenopodiaceae), a plant used in Pakistan for the treatment of several neuronal disorders, four new C-24 alkylated sterols **149 **– **152 **and five known sterols **153 **– **157 **were isolated [59]. Compounds **149 **– **157 **were analyzed as AChEi and were found to inhibit AChE in a concentration-dependent manner acting as non-competitive inhibitors. Haloxysterol B (**150**) and haloxysterol C (**151**), whose IC_50_ values were 0.89 and 1.0 µM, respectively, were found to be the most active AChE inhibitors. Their inhibitory activity was observed to be similar to that of galanthamine (IC_50_ 0.5 µM). Haloxysterol A (**149**) and 24-ethyl-cholest-6-ene-3,5-diol (**157**) were also observed to show potent AChE inhibition with IC_50_ values of 8.3 and 3.5 µM, respectively. Haloxysterol D (**152**), 5(,8(-epidioxy-(24*S*)-ethyl-cholest-6,9(11),22(*E*)-triene-3β-ol (**153**), (24*S*)-ethyl-cholest-7,9(11),22(*E*)-triene-3β-ol (**154**), lawsaritol (**155**) and 24-ethyl-cholest-7-ene-3,5,6-triol (**156**) were found to elicit a moderate anti-AChE activity (IC_50_ = 13.7 - 26.4 µM).

On the other hand, a bioassay-guided fractionation on the bark of *Mesua elegans* (Clusiaceae) allowed the isolation of the anti-AChE components responsible for the activity observed for the extract. Mesuagenin B (**158**) was the most potent inhibitor (IC_50_ = 0.7 µM) and mesuagenin A (**159**), mesuagenin D (**160**) and 5,7-dihydroxy-8-(3-methylbutanoyl)-6- (E)-3,7-dimethylocta-2,6-dienyl-4-phenyl-2H-chromen-2-one (**161**) were observed to elicit strong AChE inhibition with IC_50_ values of 1.06, 8.73 and 3.06 µM, respectively [60]. This bioassay-guided study is the first report of 4-phenylcoumarins as AChEi.

In the past, some examples of xanthones with moderate AChE inhibitory activity were reported [7]. Further recent research introduced two new xanthones, **162 **and **163**, to this group of AChEi also with moderate inhibitory activity. Macluraxanthone (**162**) which was obtained from the root of *Maclura pomifera* (Moraceae) was found to elicit non-competitive AChE inhibition (IC_50_ = 8.47 µM) [61]. Furthermore, docking studies yielded results supporting *in vitro* results. Triptexanthoside C (**163**) which was isolated from the methanolic extract of *Gentianella amarella* ssp. *acuta* (Gentianaceae) was observed to elicit AChE inhibition with an IC_50_ = 13.8 µM [62]. 

The methanolic extract of* Paulownia tormentosa *fruits, with a potent inhibitory activity against AChE, was subjected to bioactivity-guided fractionation which allowed the identification of some geranylated flavonoids, such as cholinesterase inhibitors, of which the most active resulted to be 6-geranyl-3,3’,5,5’,7-pentahydroxy-4’-methoxyflavane (**164**), 6-geranyl-3’,5,5’,7-tetrahydroxy-4’-methoxyflavanone (**165**) and diplacone (**166**), which were observed to show mixed-type inhibition of human AChE with IC_50_= 15.6, 22.9 and 7.2 µM, respectively [63]. In addition, the fact that these compounds were also observed to elicit significant BChE inhibition makes them interesting as potential dual inhibitors.

The flavonols present in *Sophora flavescens* (Fabaceae) were studied for several biological activities relevant for AD. Sophoflavescenol (**167**), icaritin (**168**), demethylanhydro-icaritin (**169**), 8-C-lavandurylkaempferol (**170**) and kaempferol (**171**) were all found to be good AChE inhibitors, with IC_50_ values of 8.37, 6.47, 6.67, 5.16 and 3.31 µM, respectively [64]. Compounds **167**-**171 **were also found to elicit significant BChE and BACE1 inhibition.

The methanol extract from roots of *Morus lhou* (Moraceae), a polyphenol-rich plant, was found to yield nine flavonoids (**172 **- **180**) of which eight showed AChE inhibition [65]. A new flavone, 5’-geranyl-4’-methoxy-5,7,2’-trihydroxyflavone (**172**), was identified as the most potent inhibitor (IC_50_ = 10.95 µM). 5’-geranyl-5,7,2’,4’-tetrahydroxyflavone (**173**), kuwanon U (**174**), kuwanon E (**175**), morusin (**176**), cyclomorusin (**178**), neocyclomorusin (**179**) and kuwanon C (**180**) were all observed to be moderate AChE inhibitors (IC_50_ = 16.21 - 36.4 µM) and morusinol (**177**) was observed to be weakly active (IC_50_ = 173.49 µM). C-3 prenylated flavones **176**,** 178**,** 179 **and **180 **were found to be noncompetitive inhibitors whereas those unsubstituted at C-3 **172**-**175 **were mixed inhibitors. Flavonoids **172 **- **180** were also found to inhibit BChE [65].

On the other hand, three potent AChEi were obtained from *Broussonetia papyrifera*, another plant belonging to the Moraceae family. From the ethanolic extract of the roots of *B. papyrifera* which was found to elicit cholinesterase inhibitory activity, prenylated flavonols **181 **– **183 **were isolated and characterized [66]. 8-(1,1-dimethylallyl)-5’-(3-methylbut-2-enyl)-3’,4’,5,7-tetrahydroxyflavonol (**181**), papyriflavonol (**182**) and broussoflavonol (**183**) were observed to inhibit human erythrocyte AChE with IC_50_ values of 0.82, 3.1 and 2.7 µM, respectively. Compound **181**, the most potent, acted as a time-dependent, slow reversible inhibitor.

Isoorientin (**184**) and isovitexin (**185**) were identified as the compounds responsible for the AChE inhibition observed in the extracts from flowers and rhizomes of *Iris pseudopumila *(Iridaceae) from Italy [67]. Compound **184 **was observed to be the highest inhibitor with an IC_50_ = 26.8 µM while **185**, lacking the 3’-hydroxy group in ring B, was observed to show an IC_50_ value of 36.4 µM. Both compounds were also found to have the ability of significantly inhibiting BChE.

On the other hand, a pterocarpan with moderate AChE inhibition was isolated from the polar extract of *Zygophyllum eurypterum* (Zygophyllaceae) collected in Pakistan. Atricarpan D [(-)-2,9-dimethoxy-4-(5-oxohexyl)pterocarpan] (**186**) was observed to inhibit AChE with an IC_50_ = 20.5 µM [68]. Interestingly, three other pterocarpans with similar structure were obtained along with atricarpan D but they were found to be inactive against AChE. Nevertheless, the four pterocarpans were all found to be BChE inhibitors.

A study conducted on AChE and BChE inhibitory activity of coumarins and naphtoquinones obtained from *Mansonia gagei* (Sterculiaceae) proposed a novel class of cholinesterase inhibitor, mansonones or 1,2-naphtoquinones [69]. The level of cholinesterase inhibition observed in this study seemed to correlate to the presence of a fused pyran ring and a substituent at C-6 being present in the molecule. Mansonone E (**187**) was observed to be the most active AChE (IC_50_ = 23.5 µM) and BChE inhibitor.

In several studies published during the period covered in the present review various phenolic compounds with different structural characteristics were reported as AChEi. Some of them are structurally simple such as gallic acid (**188**, IC_50 _= 5.85 µM) and ellagic acid (**189**, IC_50 _= 45.63 µM) [70]. Hopeahainol A (**190**), which was identified as a new compound isolated from *Hopea hainensis*, was observed to elicit a notable AChE inhibition (IC_50 _= 4.33 µM) with respect to huperzine A (IC_50 _= 1.6 µM), as a reversible mixed-type inhibitor [71].

The bioassay-guided fractionation of the extract from *Terminalia chebula* (Combretaceae) fruits allowed the isolation of 1,2,3,4,6-penta-*O*-galloyl-β-D-glucose (**191**) which demonstrated to be a significant AChE inhibitor (IC_50_ = 29.9 µM) [72]. This gallotanin which has been also isolated from other different sources and which is known by its diverse biological activities, was observed to exert good BChE inhibition and potent antioxidant activity (FRAP assay) in this study.

The bioassay-guided extraction of the stem bark of *Knema laurina* (Myristicaceae) yielded two active fractions (dichloromethane and hexane) which were subjected to chromatographic separation. That latter yielded five alkenyl phenol and salicylic acid derivatives **192 **- **196**, of which **192 **and **193 **were new compounds [73]. Compounds **192**, **195 **and **196**, all having salicylic acid moiety, were observed to strongly inhibit AChE with an IC_50_ = 3.182, 2.172 and 0.573 µM, respectively. Compounds **193 **and **194***,* with no carboxyl moiety, were observed to be good AChE inhibitors (IC_50_ = 17.224 and 13.114 µM, respectively). These findings suggest that the acidic group is key to good AChE inhibition. It was also observed that anti-AChE activity dramatically decreased when the acidic and the phenolic hydroxy group were methylated. Two catechol alkenyls were isolated from the fruits of *Semecarpus anacardium* (Anacardiaceae), a species used in Ayurvedic medicine for retarding and treatment of memory loss [74]. Compounds **197 **and **198 **were identified as active components of the dichloromethane extract through a fractionation guided by the detection of AChE inhibition. Microplate assay revealed that these catechol alkenyls are moderate and weak selective AChEi. Compound **197**, with a double bond in the aliphatic chain, was identified as a stronger inhibitor (IC_50_ = 39.7 µM) with respect to compound **198**, with two double bonds in the aliphatic chain (IC_50_ = 108 µM).

On the other hand, four structurally diverse AChEi were isolated from the polar extract of *Nelumbo nucifera* (Nelumbonaceae) stamens [75]. Cycloartenol (**199**), *p*-hydroxybenzoic acid (**200**), vanilloloside (**201**) and nuciferoside (**202**) were found to elicit good and noncompetitive inhibition against AChE with an IC_50_ = 11.89, 20.07, 4.55 and 3.2 µM, respectively. In the same study, compounds **199**, **200 **and **202 **were observed to exert moderate BChE inhibition and compounds **199 **- **202** were found to show no inhibition against BACE1.

## PLANT EXTRACTS, FRACTIONS AND ESSENTIAL OILS WITH AChE INHIBITORY ACTIVITY

Table **1** summarizes the studies published from 2006 to 2012 on plant extracts, fractions and essential oils that have been found to be good AChE inhibitors (IC_50_ < 500 µg/mL). Those plants included in other recent reviews were omitted [76, 77]. Extracts and fractions under further phytochemical studies that led to the discovery of AChE inhibitors were also omitted. Whenever possible, reference is made to the part of the plant used in each study reported. AChE inhibitory activity is reported in the same way as it was reported by authors and IC_50_ values were chosen instead of inhibition percentages when both were available.

## Figures and Tables

**Fig. (1):**
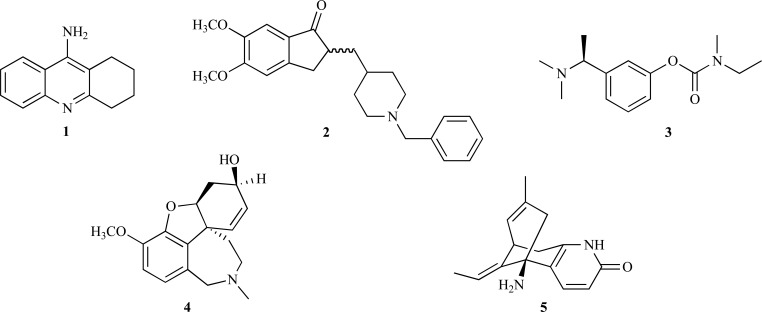


**Fig. (2):**
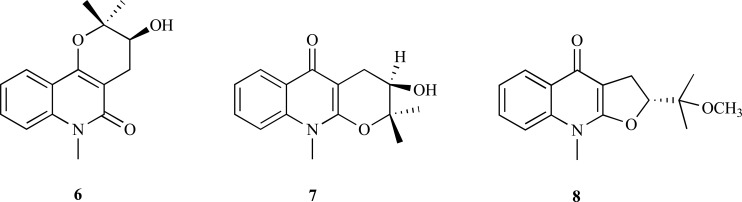


**Fig. (3):**
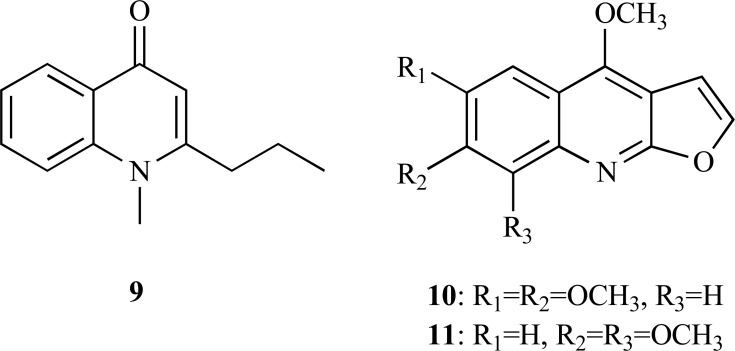


**Fig. (4):**
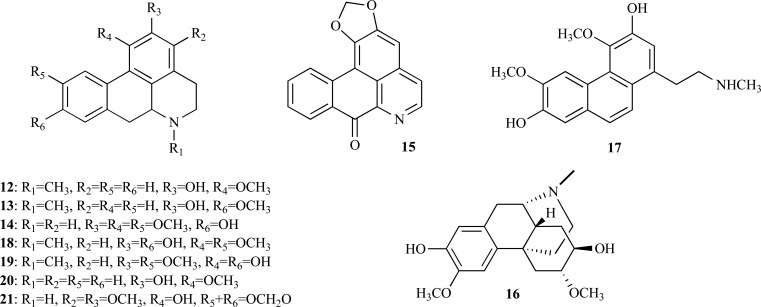


**Fig. (5):**
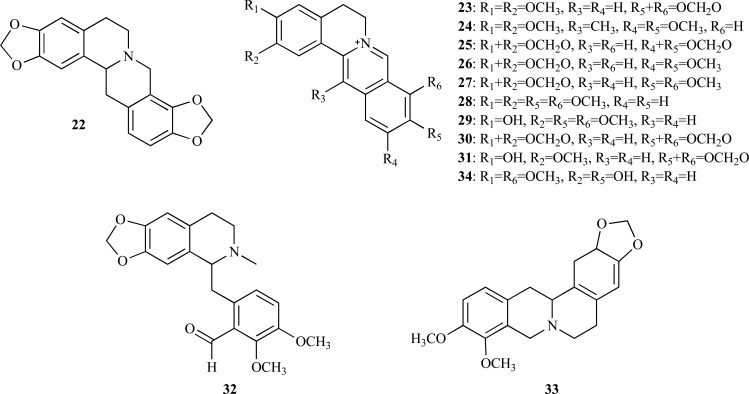


**Fig. (6):**
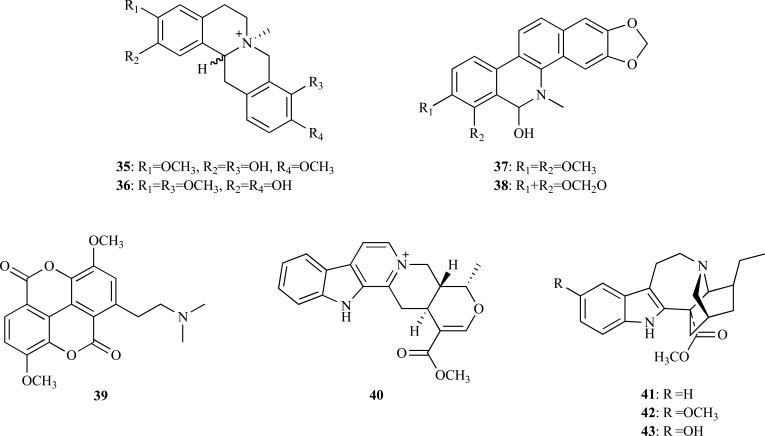


**Fig. (7):**
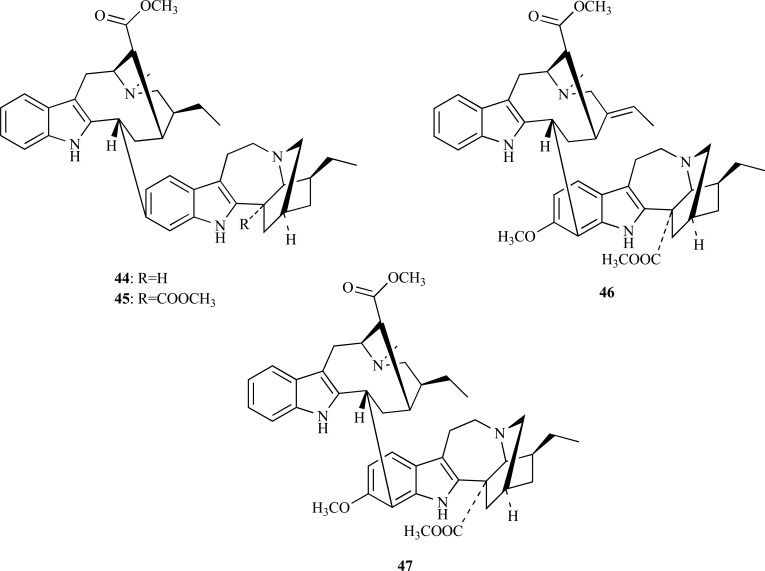


**Fig. (8):**
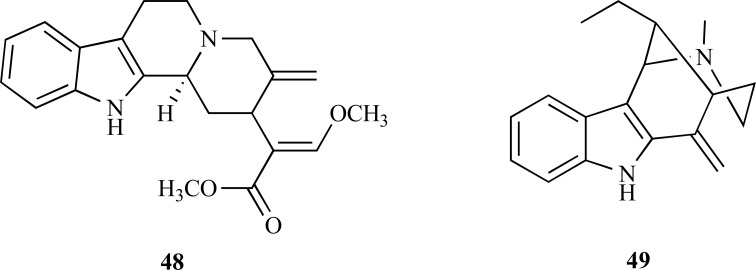


**Fig. (9):**
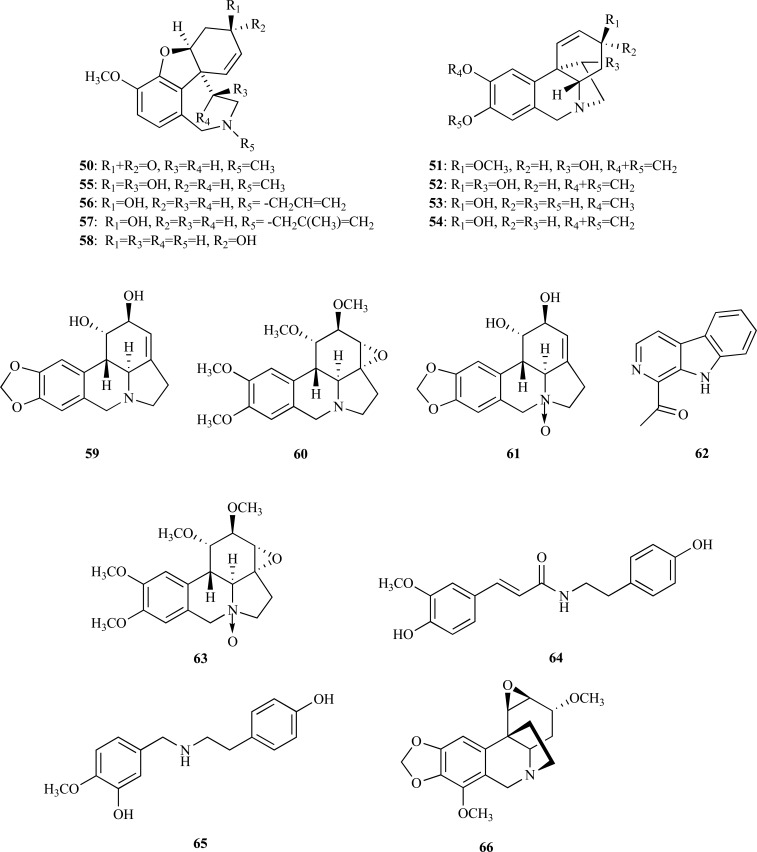


**Fig. (10):**
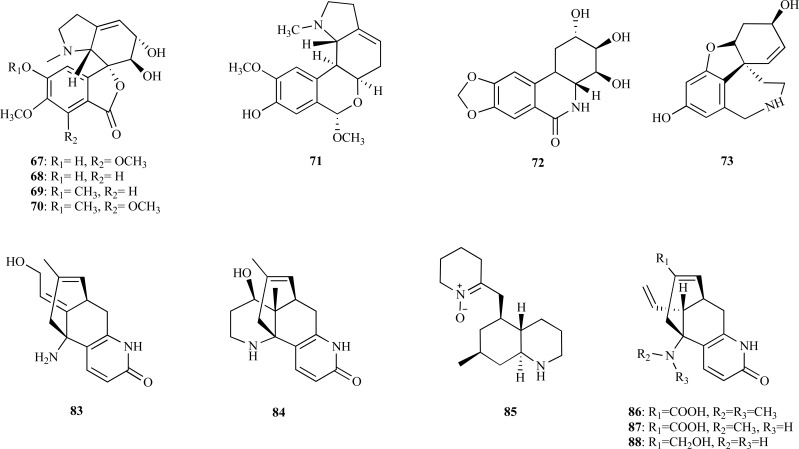


**Fig. (11):**
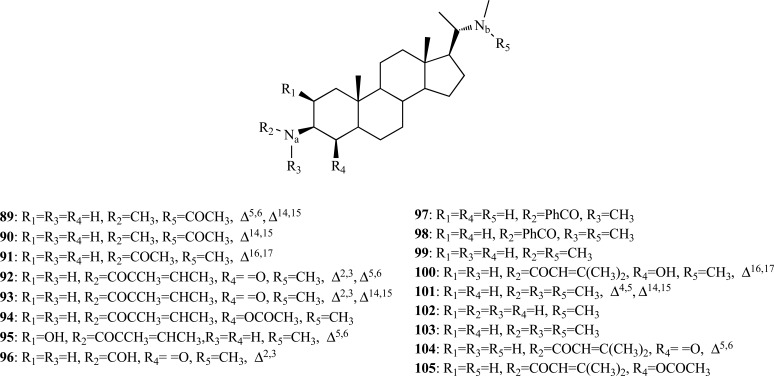


**Fig. (12):**
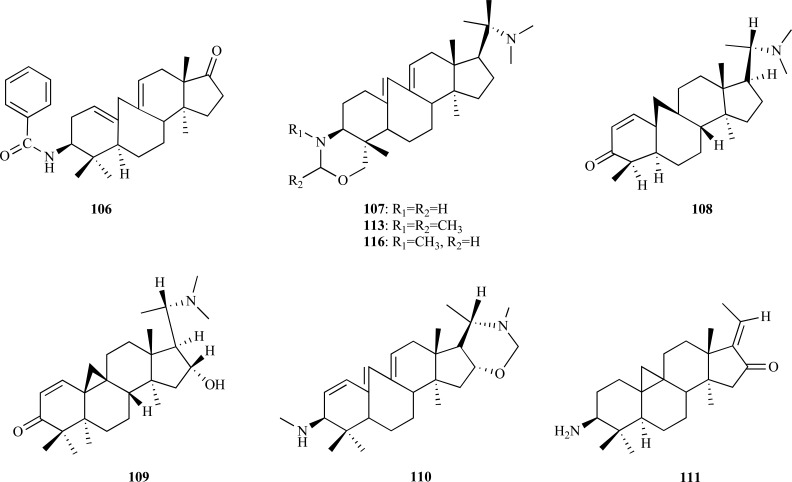


**Fig. (13):**
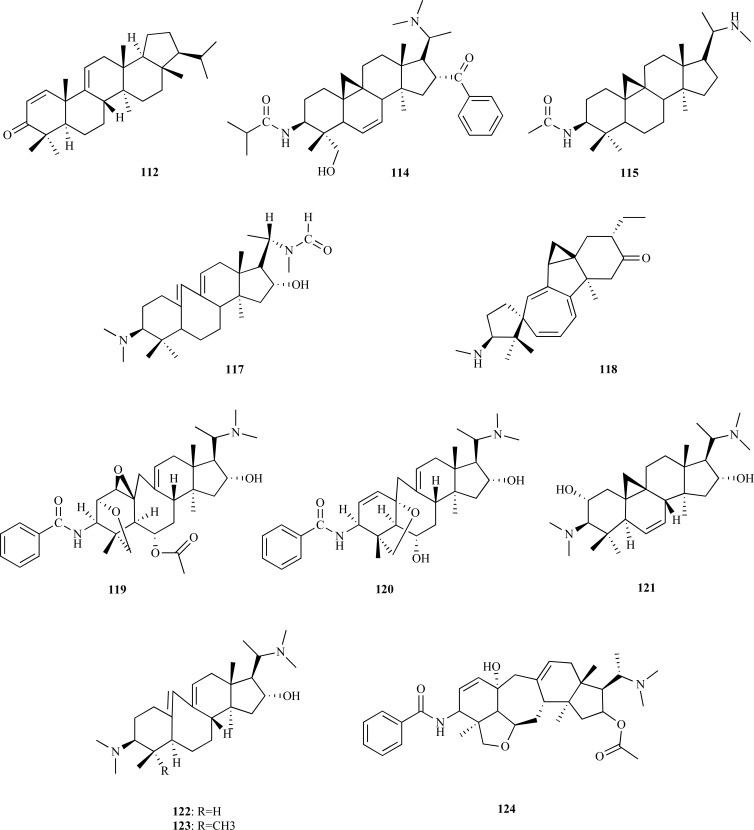


**Fig. (14):**
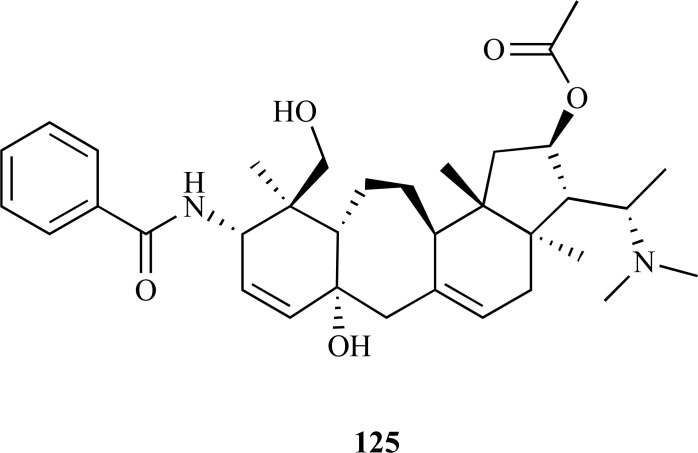


**Fig. (15):**
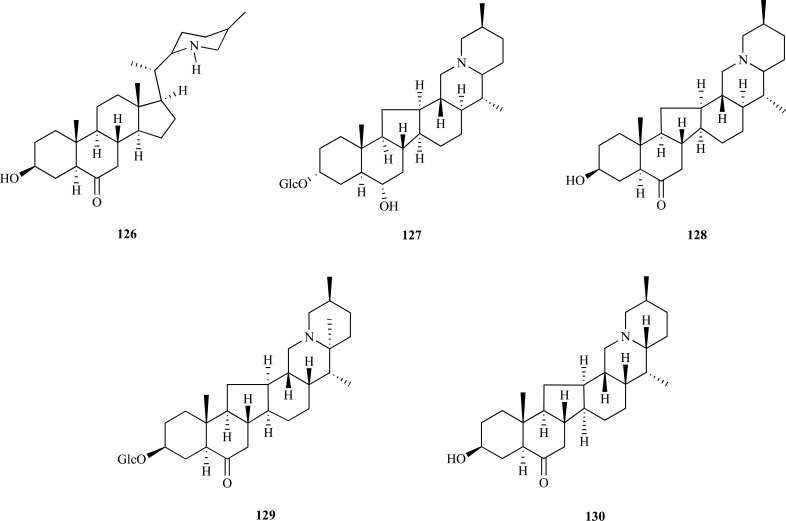


**Fig. (16):**
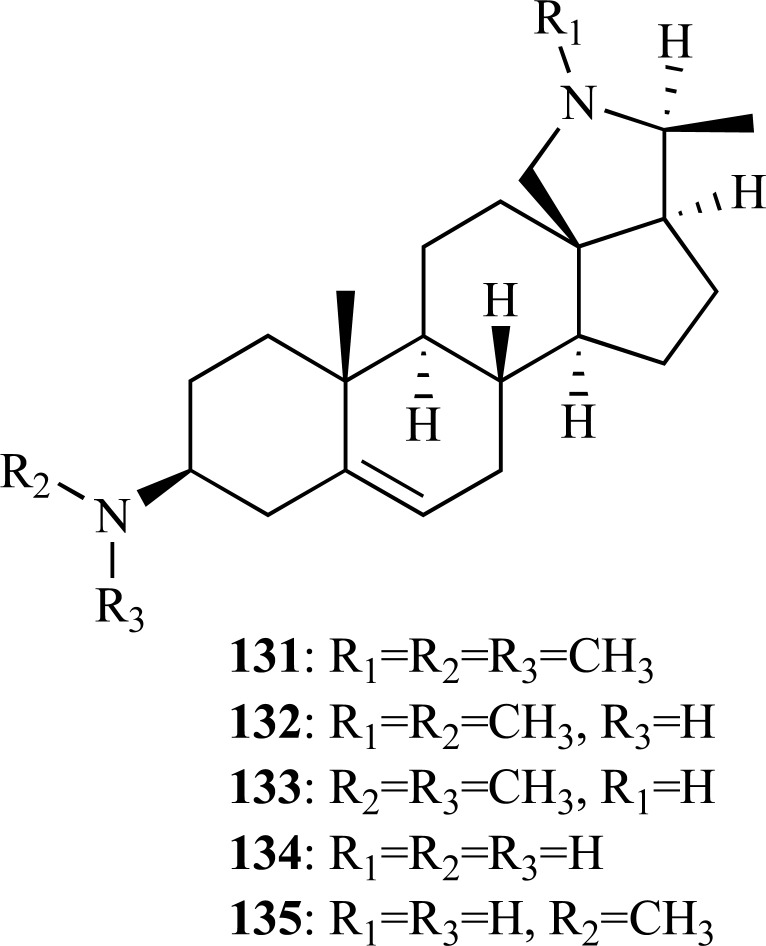


**Fig. (17):**
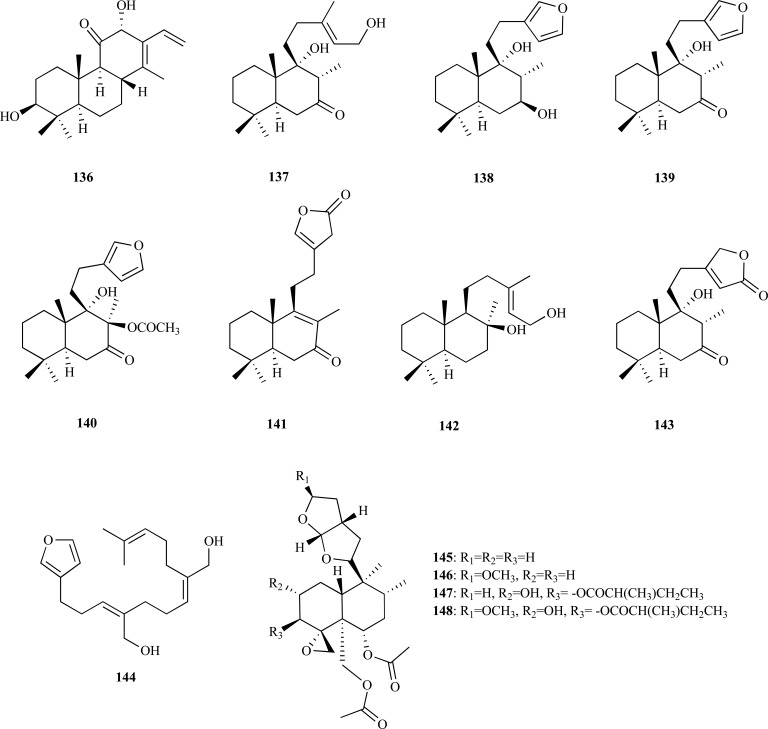


**Fig. (18):**
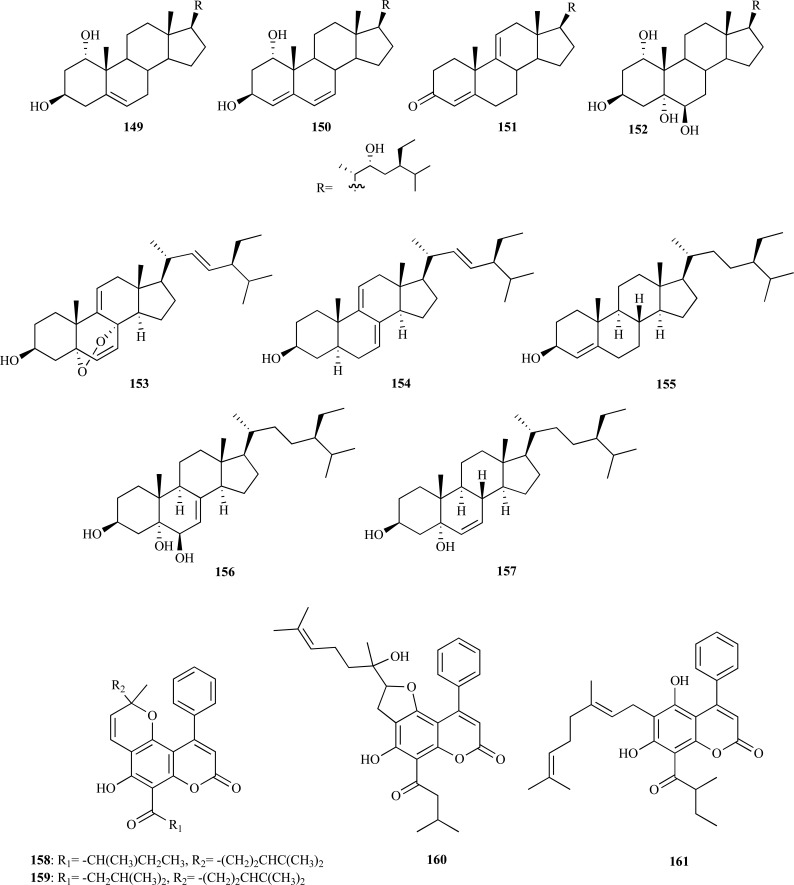


**Fig. (19):**
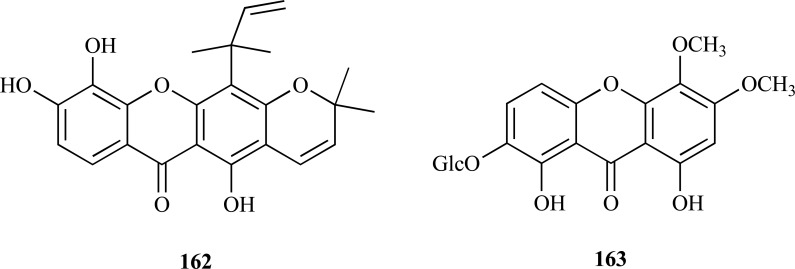


**Fig. (20):**
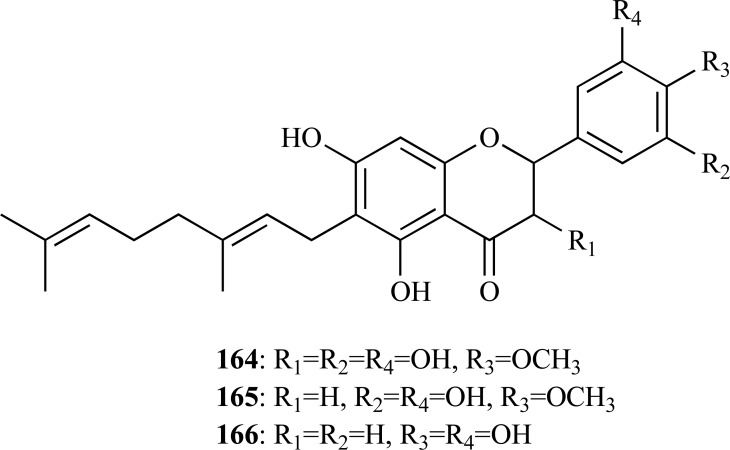


**Fig. (21):**
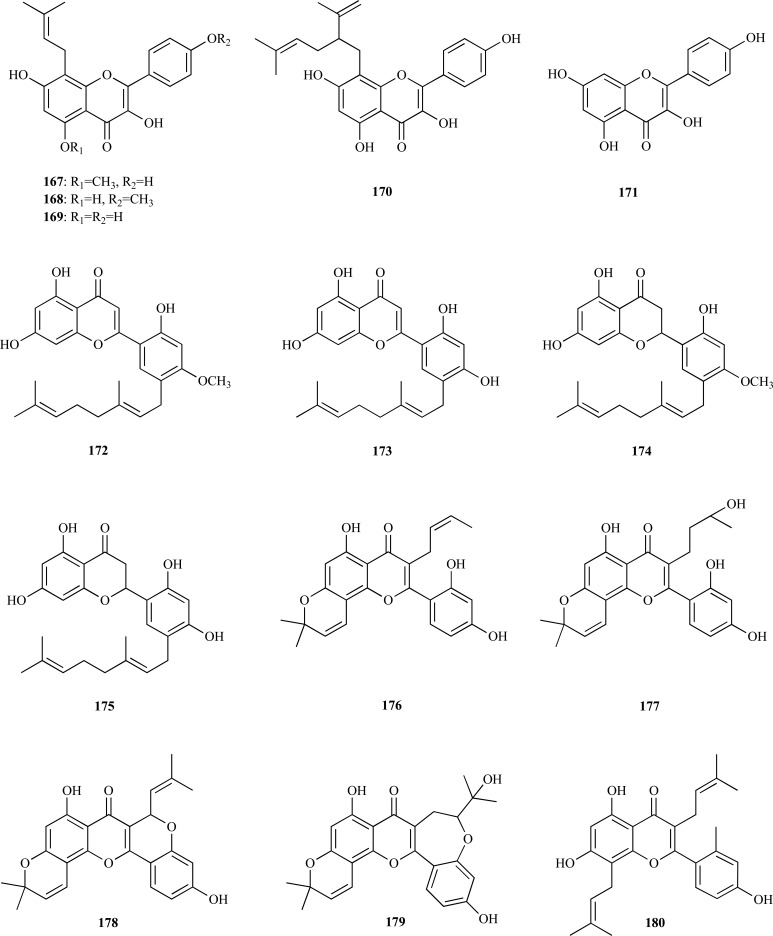


**Fig. (22):**
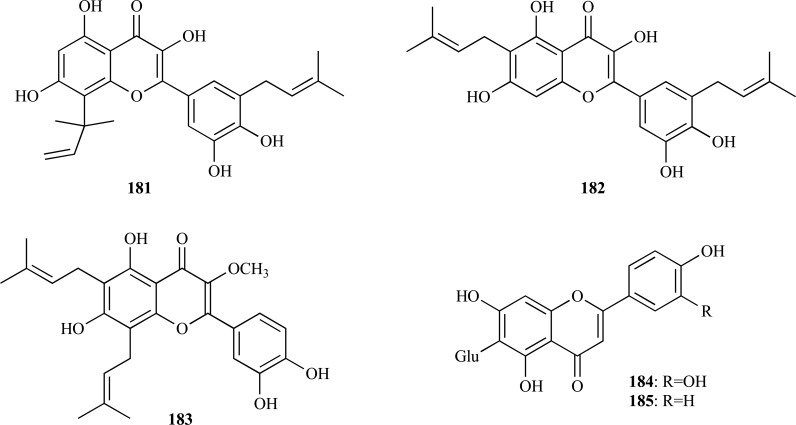


**Fig. (23):**
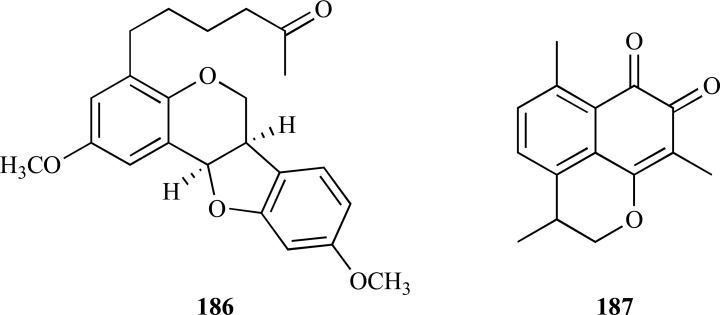


**Fig. (24):**
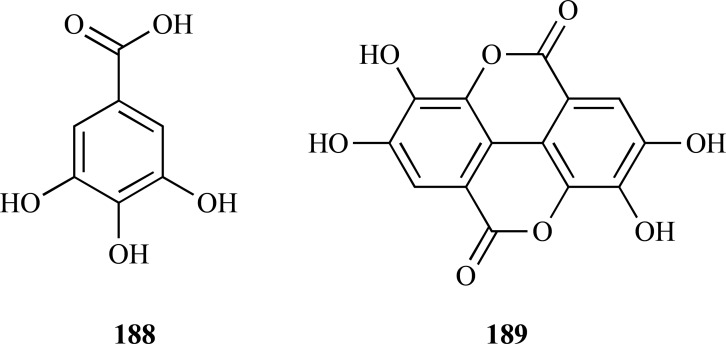


**Fig. (25):**
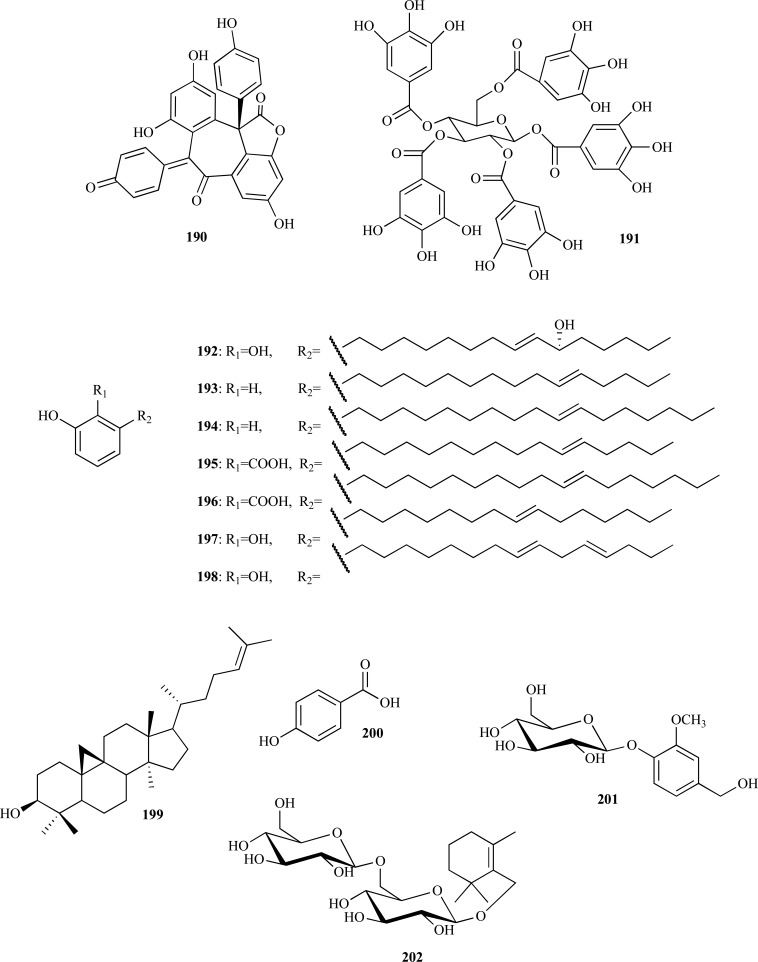


**Table 1.** Plant Extracts, Fractions and Essential Oils with AChE Inhibitory Activity

Family and Botanical Name	Type of Extract (Solvent)	Plant's Parts	AChE Inhibition (%)	IC_50_	Refs.
**Acanthaceae**					
*Andrographis paniculata *	H2O:EtOH	Aerial		222.41 µg/ml	[78]
**Amaranthaceae**					
*Salsola oppositifolia *	Alkaloids	Aerial		70.0 µg/ml	[79]
*Salsola soda *	Alkaloids	Aerial		64.1 µg/ml	[79]
*Salsola tragus*	Alkaloids	Aerial		30.2 µg/ml	[79]
**Amaryllidaceae **					
*Crinum jagus *	MeOH	Leaf	74.25 (42 µg/ml)		[80]
*Crinum moorei *	50% MeOH PE DCM EtOH	Bulb		21.5 µg/ml 18.9 µg/ml 2.9 µg/ml 22.5 µg/ml	[81]
*Nerine undulata*	Alkaloids	Bulb		14.3 µg/ml a	[82]
*Scadoxus multiflorus *	Alkaloids	Bulb		313.5 µg/ml^a^	[82]
*Sprekelia formosissima *	Alkaloids	Bulb		209.7 µg/ml^a^	[82]
*Zephyranthes grandiflora*	Alkaloids	Bulb		39.2 µg/ml^a^	[83]
**Anacardiaceae**					
*Harpephyllum caffrum*	DCM MeOH	Leaf Stem bark Leaf		0.17 mg/ml 0.02 mg/ml 0.12 mg/ml	[84]
*Pistacia atlantica *	H2O	Leaf		0.87 µg/ml	[85]
*Pistacia lentiscos *	H2O	Leaf		13.67 µg/ml	[85]
*Sclerocarya birrea*	DCM MeOH	Young stem Leaf Operculum Young stem		0.15 mg/ml 0.10 mg/ml 0.35 mg/ml 0.47 mg/ml	[84]
*Spondias mombin*	MeOH	Root bark	64.77 (42 µg/ml)		[80]
**Apiaceae**					
*Centella asiatica*	H2O:EtOH	Whole plant		106.55 µg/ml	[78]
**Apocynaceae**					
*Geissospermum vellosii*	Alkaloids	Stem bark		2.9 µg/ml	[86]
**Araceae**					
*Colocasia antiquorum*	50% MeOH PE DCM	Tuber		7.9 µg/ml 6.4 µg/ml 168.1 µg/ml	[81]
*Pinellia ternata*	Alkaloids	Tuber		56.2 µg/ml	[87]
**Arecaceae**					
*Phoenix dactylifera*	Hexane	Seed	52.96 (300 µg/ml)		[88]
**Asparagaceae**					
*Leopoldia comosa *	Hexane	Bulb		104.9 µg/ml	[89]
**Asphodelaceae**					
*Aloe ferox *	50% MeOH PE DCM	Leaf		84.0 µg/ml 37.7 µg/ml 62.6 µg/ml	[81]
**Asteraceae**					
*Achyrocline tomentosa*	Organic	Aerial		0.4847 mg/ml	[90]
*Arnica chamissonis *ssp. *foliosa *	MeOH Hexane	Flower		43 µg/ml 29 µg/ml	[91]
*Chromolaena tequendamensis *	MeOH	Whole plant		359.36 mg/l	[92]
*Eupatorium viscidum*	Organic	Aerial		0.4792 mg/ml	[90]
*Pulicaria stephanocarpa*	CHCl3	Leaf	61.43 (0.2 mg/ml)		[93]
*Schistocarpha sinforosi *	MeOH	Whole plant		145.31 mg/l	[92]
*Trichocline reptans*	Organic	Aerial		0.1118 mg/ml	[90]
**Berberidaceae**					
*Berberis darwinii*	MeOH	Stem bark		1.23 µg/ml	[94]
**Boraginaceae**					
*Onosma bracteata*	MeOH	Leaf	59.73 (250 µg/ml)		[95]
**Buddlejaceae**					
*Buddleja salviifolia *	DCM:MeOH (1:1)	Whole plant		0.05 mg/ml	[96]
**Burseraceae**					
*Boswellia socotranao *	CHCl3	Resin	71.21 (0.2 mg/ml)		[93]
**Cistaceae**					
*Cistus laurifolius *	EtOH	Leaf	80.07 (200 µg/ml)		[97]
**Combretaceae**					
*Terminalia bellirica*	MeOH	Fruit		14.37 µg/ml	[70]
**Convolvulaceae**					
*Evolvulus alsinoides*	H2O:EtOH	Whole plant		141.76 µg/ml	[78]
*Ipomoea asarifolia*	MeOH	Leaf		0.12 µg/ml	[98]
**Crassulaceae**					
*Kalanchoe brasiliensis *	EtOAc	Leaf		0.16 mg/ml	[98]
**Cucurbitaceae**					
*Eureiandra balfourii *	MeOH	Tuber	58.61 (0.2 mg/ml)		[93]
**Cupressaceae**					
*Juniperus phoenicea*	EtOH	Leaf	53.44 (400 µg/ml)		[99]
*Juniperus turbinata*	Phenolic	Leaf	83.84 (400 µg/ml)		[99]
**Ericaceae**					
*Rhododendron yedoense *var.* poukhanense *	80% MeOH	Bark		169.01 µg/ml	[100]
**Eucommiaceae**					
*Eucommia ulmoides *	H2O	Bark		172 µg/ml	[101]
**Euphorbiaceae**					
*Alchornia laxiflora *	MeOH	Stem bark	41.12 (42 µg/ml)		[80]
*Cephalocroton socotranus *	CHCl3	Bark	51.1 (0.2 mg/ml)		[93]
*Jatropha curcas *	MeOH	Leaf		0.25 mg/ml	[98]
*Jatropha gossypiifolia *	MeOH	Leaf		0.05 mg/ml	[98]
**Fabaceae**					
*Acacia nilotica *	H2O	Root		0.079 mg/ml^b^	[102]
*Acacia raddiana*	H2O	Bark		33.91 µg/ml	[85]
*Cassia obtusifolia*	EtOH	Seed		81.6 µg/ml c	[103]
*Chamaecrista mimosoides*	DCM:MeOH (1:1) H2O	Root		0.03 mg/ml 0.35 mg/ml	[96]
*Genista tenera*	EtOAc	Aerial	77.0 (70 µg/ml)		[105]
*Peltophorum pterocarpum *	MeOH	Leaf Stem bark	49.5 (42 µg/ml) 68.85 (42 µg/ml)		[80]
*Schotia brachypetala *	DCM:MeOH (1:1) H2O	Bark		0.27 mg/ml 0.49 mg/ml	[96]
*Senna alata *	EtOAc	Leaf		0.08 mg/ml	[98]
*Spatholobus suberectus*	H2O EtOH	Whole plant		85 µg/ml 9 µg/ml	[104]
*Trigonella foenum-graecum*	EtOAc Alkaloids	Seed		53.00 µg/ml 9.23 µg/ml	[106]
**Gobulariaceae **					
*Globularia alypum*	H2O	Root		16.67 µg/ml	[85]
**Guttiferaceae**					
*Callophyllum inophyllurn *	MeOH	Root bark	56.52 (42 µg/ml)		[80]
**Hypericaceae**					
*Hypericum perforatum *	MeOH	Whole plant		178 µg/ml	[91]
**Illiciaceae**					
*Illicium verum*	H2O:EtOH Butanol EtOAc CHCl3 Oil	Fruit		58.67 µg/ml 44.94 µg/ml 83.75 µg/ml 103.03 µg/ml 39.89 µg/ml	[107]
**Lamiaceae**					
*Cyclotrichium niveum*	EtOAc DCM	Whole plant	83.11 (250 µg/ml) 70.82 (250 µg/ml)		[108]
*Hyssopus officinials *	Hexane	Whole plant	55.0 (400 µg/ml)		[91]
*Lavandula viridis *	MeOH	Aerial		244.55 µg/ml	[109]
*Marrubium vulgare*	Acetone	Aerial	62.70 (25 µg/ml)		[110]
*Origanum ehrenbergii*	Essential oil	Aerial		0.3 µg/ml	[111]
*Origanum majorana *	Essential oil	Leaf		36.40 µg/ml	[112]
*Origanum syriacum*	Essential oil	Aerial		1.7 µg/ml	[111]
*Pycnostachys reticulata *	50% MeOH EtOH	Leaf		28.8 µg/ml 8.8 µg/ml	[81]
*Salvia chionantha*	Essential oil	Aerial	56.7 (500 µg/ml)		[113]
*Salvia fruticosa *	DCM	Whole plant	51.07 (100 µg/ml)		[114]
*Salvia leriifolia*	Essential oil	Aerial		0.32 µl/ml	[115]
*Salvia miltiorrhiza*	H2O EtOH	Root		50 µg/ml 5 µg/ml	[104]
*Teucrium royleanum*	MeOH	Whole plant	52.4 (40 µg/0.2ml)		[116]
**Menispermaceae**					
*Stephania pierrei*	EtOH	Tuber		5.68 µg/ml	[117]
*Tinospora cordifolia *	MeOH	Stem		38.36 µg/ml	[118]
**Moraceae **					
*Dorstenia gigas*	CHCl3	Leaf	65.12 (0.2 mg/ml)		[93]
*Ficus religiosa*	MeOH	Stem bark		73.69 µg/ml	[118]
**Myristicaceae**					
*Myristica fragrans*	H2O:EtOH	Seed		133.28 µg/ml	[78]
*Embelia ribes *	MeOH	Root		23.04 µg/ml	[118]
**Orchidaceae**					
*Orchis mascula *	MeOH	Root	56.99 (250 µg/ml)		[119]
**Paeoniaceae**					
*Paeonia lactiflora *	H2O EtOH	Root		20 µg/ml 8 µg/ml	[104]
*Paeonia veitchii*	H2O EtOH	Root		14 µg/ml 45 µg/ml	[104]
**Papaveraceae**					
*Corydalis intermedia*	MeOH H2O	Whole plant Tuber Whole plant Tuber	84 (100 µg/ml) 97 (100 µg/ml) 57 (100 µg/ml) 78 (100 µg/ml)		[120]
**Papaveraceae**					
*Corydalis solida *ssp. laxa	MeOH H2O	Whole plant Tuber Whole plant Tuber	89 (100 µg/ml) 96 (100 µg/ml) 78 (100 µg/ml) 85 (100 µg/ml)		[120]
*Corydalis solida *ssp. slivenensis	MeOH H2O	Whole plant Tuber Whole plant Tuber	82 (100 µg/ml) 97 (100 µg/ml) 48 (100 µg/ml) 87 (100 µg/ml)		[120]
**Phyllantaceae**					
*Emblica officinalis*	MeOH	Fruit		29.26 µg/ml	[70]
**Pinaceae**					
*Pinus halepensis*	EtOH Essential oil	Needle Twig	60.15 (200 µg/ml) 83.91 (200 µg/ml)		[121]
*Pinus heldreichii *subsp. *leucodermis*	Essential oil	Needle		51.1 µg/ml	[122]
*Pinus nigra *subsp.* nigra *	Essential oil	Needle		94.4 µg/ml	[122]
*Pinus nigra *subsp.* calabrica *	Essential oil	Needle		101.5 µg/ml	[122]
*Pinus pinaster*	Pycnogenol	Bark	63.33 (200 µg/ml)		[121]
**Piperaceae**					
*Piper nigrum *	EtOH	Fruit		30.67 µg/ml	[117]
**Poaceae **					
*Cymbopogon jawarancusa*	MeOH	Whole plant	72.36 (250 µg/ml)		[95]
*Cymbopogon schoenanthus *	Essential oil	Fresh leaf (mountain reg./ desert reg.) Dried leaf (mountain reg./ desert reg.) Dried root (mountain reg./ desert reg.)		0.26 / 0.67 mg/ml 0.44 / 0.52 mg/ml 0.27 / 0.32 mg/ml	[123]
*Cymbopogon schoenanthus *	Hexane DCM EtOAc MeOH H2O	Shoot (mountain reg./ desert reg.)		0.50 / 0.54 mg/ml 0.57 / 0.30 mg/ml 0.23 / 0.30 mg/ml 0.23 / 0.25 mg/ml 0.46 / 0.04 mg/ml	[124]
**Polygonaceae**					
*Fallopia multiflora *	H2O EtOH	Root		13 µg/ml 65 µg/ml	[104]
*Rheum palmatum*	H2O EtOH	Root and Rizhome		32 µg/ml 18 µg/ml	[104]
*Ruprechtia apetala*	EtOH	Aerial		0.0779 mg/ml	[90]
**Portulacaceae**					
*Portulaca oleracea*	Alkaloids	Upper part		29.4 µg/ml	[87]
**Rhamnaceae**					
*Rhamnus prinoides*	H2O	Root		0.201 mg/ml^b^	[102]
**Rosaceae**					
*Leucosidea sericea*	PE DCM MeOH PE	Leaf Stem		0.16 mg/ml 0.14 mg/ml 0.24 mg/ml 0.26 mg/ml	[125]
**Rubiaceae**					
*Galium odoratum*	Hexane	Whole plant	53.1 (400 µg/ml)		[91]
*Morinda citrifolia*	EtOH CHCl3	Fruit		138.4 µg/ml 78.11 µg/ml	[126]
*Morinda lucida*	MeOH	Leaf	40.15 (42 µg/ml)		[80]
**Rutaceae**					
*Citrus aurantifolia *	Essential oil	Leaf		139 µg/ml	[127]
*Citrus medica*	Essential oil	Peel		171.3 µg/ml	[128]
*Ruta graveolens *	MeOH Hexane	Whole plant	59.1 (100 µg/ml)	34 µg/ml	[91]
*Zanthoxylum coco *	Organic	Aerial		0.1579 mg/ml	[90]
**Solanaceae**					
*Solanum leucocarpum *	MeOH	Whole plant		204.59 mg/l	[92]
*Withania somnifera *	MeOH	Root		33.38 µg/ml	[118]
*Witheringia coccoloboides *	MeOH	Whole plant		220.68 mg/l	[92]
**Valerianaceae**					
*Nardostachys jatamansi*	H2O:EtOH MeOH	Rhizome		130.11 µg/ml 47.21 µg/ml	[78] [118]
**Zingiberaceae **					
*Kaempfera parviflora*	EtOH	Rhizome		20.64 µg/ml	[117]

DCM: dichloromethane; MeOH: methanol; EtOH: ethanol; PE: petroleum ether; EtOAc: ethyl acetate

a Human blood AChE.

b Bovine erythrocyte AChE.

c Mouse brain homogenized.

## References

[1] Chopra K, Misra S, Kuhad A (2011). Current perspectives on pharmacotherapy of Alzheimer’s. Expert. Opin. Pharmacother..

[2] Ellman GL, Courtney KD, Andres V, Featherstone RM (1961). A new and rapid colorimetric determination of acetylcholinesterase activity. Biochem. Pharmacol.

[3] Lopez S, Bastida J, Viladomat F, Codina C (2002). Acetylcholinesterase inhibitory activity of some Amaryllidaceae alkaloids and Narcissus extracts. Life Sci..

[4] Rhee IK, van de Meent M, Ingkaninan K, Verpoorte R (2001). Screening for acetylcholinesterase inhibitors from Amaryllidaceae using silica gel thin-layer chromatography in combinatioin with bioactivity staining. J. Chromatogr. A.

[5] Marston A, Kissling J, Hostettmann K (2002). A rapid TLC bioautographic method for the detection of acetylcholinesterase and butyrylcholinesterase inhibitors in plants. Phytochem. Anal..

[6] Di Giovanni S, Borloz A, Urbain A, Marston A, Hostettmann K, Carrupt PA, Reist M (2008). In vitro screening assays to identify natural or synthetic acetylcholinesterase inhibitors: thin layer chromatography versus microplate methods. Eur. J. Pharm. Sci..

[7] Houghton PJ, Ren Y, Howes MJ (2006). Acetylcholinesterase inhibitors from plants and fungi. Nat. Prod. Rep..

[8] Williams P, Sorribas A, Howes MJ (2011). Natural Products as a source of Alzheimer’s drugs leads. Nat. Prod. Rep..

[9] Mukherjee PK, Kumar V, Mal M, Houghton PJ (2007). Acetylcholinesterase inhibitors from plants. Phytomedicine.

[10] Orhan G, Orhan I, Subutay-Oztekin N, Ak F, Sener B (2009). Contemporary anticholinesterase pharmaceuticals of natural origin and their synthetic analogues for the treatment of Alzheimer's disease. Recent. Pat. CNS Drug. Discov..

[11] Rahman AU, Khalid A, Sultana N, Ghayur MN, Mesaik MA, Khan MR, Gilani AH, Choudhary MI (2006). New natural cholinesterase inhibiting and calcium channel blocking quinoline alkaloids. J. Enzyme Inhib. Med. Chem..

[12] Cardoso-Lopes EM, Maier A, da Silva MR, Regasini LO, Simote SY, Lopes NP, Pirani JR, da Silva Bolzani V, Marx Young MC (2010). Alkaloids from stems of Esenbeckia leiocarpa Engl.(Rutaceae) as potential treatment for Alzheimer Disease. Molecules.

[13] Yang Z, Zhang D, Ren J, Yang M (2012). Skimmianine, a furoquinoline alkaloid from Zanthoxylum nitidum as a potential acetylcholinesterase inhibitor. Med. Chem. Res..

[14] Mukherjee PK, Mukherjee D, Maji AK, Rai S, Heinrich M (2009). The sacred lotus (Nelumbo nucifera) - phytochemical and therapeutic profile. J. Pharm. Pharmacol..

[15] Yang Z, Zhang X, Du J, Ma ZJ, Guo F, Li S, Yao XJ (2012). An aporphine alkaloid from Nelumbo nucifera as an acetylcholinesterase inhibitor and the primary investigation for structure-activity correlations. Nat. Prod. Res..

[16] Mollataghi A, Coudiere E, Hadi AH, Mukhtar MR, Awang K, Litaudon M, Ata A (2012). Anti-acetylcholinesterase, anti-a-glucosidase, anti-leishmanial and anti-fungal activities of chemical constituents of Beilschmiedia species. Fitoterapia.

[17] Hung TM, Na M, Dat NT, Ngoc TM, Youn U, Kim HJ, Min BS, Lee J, Bae K (2008). Cholinesterase inhibitory and anti-amnesic activity of alkaloids from Corydalis turtschaninovii. J. Ethnopharmacol..

[18] Hung TM, Ngoc TM, Youn UJ, Min BS, Na M, Thuong PT, Bae K (2008). Anti-amnesic activity of pseudocoptisine from Corydalis tuber. Biol. Pharm. Bull..

[19] Jung HA, Min BS, Yokozawa T, Lee JH, Kim YS, Choi JS (2009). Anti-Alzheimer and antioxidant activities of Coptidis Rhizoma alkaloids. Biol. Pharm. Bull..

[20] Chlebek J, Macáková K, Cahlíkovi L, Kurfürst M, Kunes J, Opletal L (2011). Acetylcholinesterase and butyrylcholinesterase inhibitory compounds from Corydalis cava (Fumariaceae). Nat. Prod. Commun..

[21] Ingkaninan K, Phengpa P, Yuenyongsawad S, Khorana N (2006). Acetylcholinesterase inhibitors from Stephania venosa tuber. J. Pharm. Pharmacol..

[22] Cho KM, Yoo ID, Kim WG (2006). 8-Hydroxydihydrochelerythrine and 8-Hydroxydihydrosanguinarine with a potent acetylcholinesterase inhibitory activity from Chelidonium majus L. Biol. Pharm. Bull..

[23] Rollinger JM, Schuster D, Baier E, Ellmerer EP, Langer T, Stuppner H (2006). Taspine bioactivity-guided isolation and molecular ligand-target insight of a potent acetylcholinesterase inhibitor from Magnolia x soulangiana. J. Nat. Prod..

[24] Pereira DM, Ferreres F, Oliveira JM, Gaspar L, Faria J, Valentão P, Sottomayor M, Andrade PB (2010). Pharmacological effects of Catharanthus roseus root alkaloids in acetylcholinesterase inhibition and cholinergic neurotransmission. Phytomedicine.

[25] Zhan ZJ, Yu Q, Wang ZL, Shan WG (2010). Indole alkaloids from Ervatamia hainanensis with potent acetylcholinesterase inhibition activities. Bioorg. Med. Chem. Lett..

[26] Andrade MT, Lima JA, Pinto AC, Rezende CM, Carvalho MP, Epifanio RA (2005). Indole alkaloids from Tabernaemontana australis (Muell.Arg) Miers that inhibit acetylcholinesterase enzyme. Bioorg. Med. Chem..

[27] Ingkaninan K, Changwijit K, Suwanborirux K (2006). Vobasinyl-iboga bisindole alkaloids, potent acetylcholinesterase inhibitors from Tabernaemontana divaricata root. J. Pharm. Pharmacol..

[28] Yang Z, Duan DZ, Du J, Yang MJ, Li S, Yao XJ (2012). Geissoschizine methyl ether, a corynanthean-type indole alkaloid from Uncaria rhynchophylla as a potential acetylcholinesterase inhibitor. Nat. Prod. Res..

[29] Seidl C, Correia BL, Stinghen AE, Santos CA (2010). Acetylcholinesterase inhibitory activity of uleine from Himatanthus lancifolius. Z. Naturforsch. C..

[30] Jin Z (2007). Amaryllidaceae and Sceletium alkaloids. Nat. Prod. Rep..

[31] de Andrade JP, Berkov S, Viladomat F, Codina C, Zuanazzi JA, Bastida J (2011). Alkaloids from Hippeastrum papillo. Molecules.

[32] Berkov S, Codina C, Viladomat F, Bastida J (2008). N-Alkylated galanthamine derivatives: Potent acetylcholinesterase inhibitors from Leucojum aestivum. Bioorg. Med. Chem. Lett..

[33] Sarikaya BB, Kaya GI, Onur MA, Viladomat F, Codina C, Bastida J, Somer NU (2012). Alkaloids from Galanthus rizehensis. Phytochem. Lett..

[34] Rijn RM, Rhee IK, Verpoorte R (2010). Isolation of acetylcholinesterase inhibitory alkaloids from Nerine bowdenii. Nat. Prod. Res..

[35] Mulholland DA, Crouch N, Decker B, Smith M T (2002). The isolation of the Amaryllidaceae alkaloid crinamine from Dioscorea dregeana. Biochem. Syst. Ecol..

[36] Wang YH, Zhang ZK, Yang FM, Sun QY, He HP, Di YT, Mu SZ, Lu Y, Chang Y, Zheng QT, Ding M, Dong JH, Hao XJ (2007). Benzylphenethylamine Alkaloids from Hosta plantaginea with Inhibitory Activity against Tobacco Mosaic Virus and Acetylcholinesterase. J. Nat. Prod..

[37] Ma X, Gang DR (2004). The Lycopodium alkaloids. Nat. Prod. Rep..

[38] Katakawa K, Nozoe A, Kogure N, Kitajima M, Hosokawa M, Takayama H (2007). Fawcettimine-related alkaloids from Lycopodium serratum. J. Nat. Prod..

[39] Takayama H, Katakawa K, Kitajima M, Yamaguchi K, Aimi N (2003). Ten new Lycopodium alkaloids having the lycopodane skeleton isolated from Lycopodium serratum Thunb. Chem. Pharm. Bull. (Tokyo).

[40] Choo CY, Hirasawa Y, Karimata C, Koyama K, Sekiguchi M, Kobayashi J, Morita H (2007). Carinatumins A–C, new alkaloids from Lycopodium carinatum inhibiting acetylcholinesterase. Bioorg. Med. Chem..

[41] Hirasawa Y, Kato E, Kobayashi J, Kawahara N, Goda Y, Shiro M, Morita H (2008). Lycoparins A-C, new alkaloids from Licopodium casuarinoides inhibiting acetylcholinesterase. Bioorg. Med. Chem..

[42] Howes M-JR, Houghton PJ (2009). Acetylcholinesterase inhibitors of natural origin. Int. J. Biomed. Pharm. Sci..

[43] Devkota KP, Lenta BN, Fokou PA, Sewald N (2008). Terpenoid alkaloids of the Buxaceae family with potential biological importance. Nat. Prod. Rep..

[44] Rahman AU, Ul-Haq Z, Khalid A, Anjum S, Khan MR, Choudhary MI (2002). Pregnane-Type steroidal alkaloids of Sarcococca saligna: a new class of cholinesterase inhibitors. Helv. Chim. Acta.

[45] Rahman AU, Feroz F, Ul-Haq Z, Nawaz SA, Khan MR, Choudhary MI (2003). New steroidal alkaloids from Sarcococca saligna. Nat. Prod. Res..

[46] Rahman AU, Feroz F, Naeem I, Ul-Haq Z, Nawaz SA, Khan N, Khan MR, Choudhary MI (2004). New pregnane-type steroidal alkaloids from Sarcococca saligna and their cholinesterase inhibitory activity. Steroids Source.

[47] Devkota KP, Lenta BN, Choudhary MI, Naz Q, Fekam FB, Rosenthal PJ, Sewald N (2007). Cholinesterase inhibiting and antiplasmodial steroidal alkaloids from Sarcococca hookeriana. Chem. Pharm. Bull. (Tokyo).

[48] Devkota KP, Lenta BN, Wansi J, Choudhary MI, Kisangau DP, Naz Q, SamreenSewald N (2008). Bioactive 5?-Pregnane-type steroidal alkaloids from Sarcococca hookeriana. J. Nat. Prod..

[49] Ata A In Studies in Natural Products Chemistry 1st Edition, Vol.38 Rahman A.

[50] Ata A, Iverson CD, Kalhari KS, Akhter S, Betteridge J, Meshkatalsadat MH, Orhan I, Sener B (2010). Triterpenoidal alkaloids from Buxus hyrcana and their enzyme inhibitory, anti-fungal and anti-leishmanial activities. Phytochemistry.

[51] Matochko WL, James A, Lam CW, Kozera D, Ata A, Gengan R (2010). Triterpenoidal alkaloids from Buxus natalensis and their acetylcholinesterase inhibitory activity. J. Nat. Prod..

[52] Rahman AU, Akhtar MN, Choudhary MI, Tsuda Y, Sener B, Khalid A, Parvez M (2002). New steroidal alkaloids from Fritillaria imperialis and their cholinesterase inhibiting activities. Chem. Pharm. Bull..

[53] Lin BQ, Ji H, Li P, Fang W, Jiang Y (2006). Inhibitors of acetylcholine esterase in vitro - Screening of steroidal alkaloids from Fritillaria species. Planta Med..

[54] Yang Z, Duan DZ, Xue WW, Yao XJ, Li S (2012). Steroidal alkaloids from Holarrhena antidysenterica as acetylcholinesterase inhibitors and the investigation for structure–activity relationships. Life Sci..

[55] Eldeen IM, VanHeerden FR, VanStaden J (2010). In vitro biological activities of niloticane, a new bioactive cassane diterpene from the bark of Acacia nilotica subsp. kraussiana. J. Ethnopharmacol..

[56] Hung TM, Luan TC, Vinh BT, Cuong TD, Min BS (2011). Labdane-type diterpenoids from Leonurus heterophyllus and their cholinesterase inhibitory activity. Phytother. Res..

[57] Khan I, Nisar M, Khan N, Saeed M, Nadeem S, Fazal-ur-Rehman; Ali F, Karim N, Kaleem WA, Qayum M, Ahmad H, Khan IA (2010). Structural insights to investigate Conypododiol as a dual cholinesterase inhibitor from Asparagus adscendens. Fitoterapia.

[58] Riaz N, Nawaz SA, Mukhtar N, Malik A, Afza N, Ali S, Ullah S, Muhammad P, Choudhary MI (2007). Isolation and enzyme-inhibition studies of the chemical constituents from Ajuga bracteosa. Chem. Biodivers..

[59] Ahmed E, Nawaz SA, Malik A, Choudhary MI (2006). Isolation and cholinesterase-inhibition studies of sterols from Haloxylon recurvum. Bioorg. Med. Chem. Lett..

[60] Awang K, Chan G, Litaudon M, Ismail NH, Martin MT, Gueritte F (2010). 4-Phenylcoumarins from Mesua elegans with acetylcholinesterase inhibitory activity. Bioorg. Med. Chem..

[61] Khan MT, Orhan I, Senol FS, Kartal M, Sener B, Dvorská M, Smejkal K, Slapetová T (2009). Cholinesterase inhibitory activities of some flavonoid derivatives and chosen xanthone and their molecular docking studies. Chem. Biol. Interact..

[62] Urbain A, Marston A, Sintro Grilo L, Bravo J, Purev O, Purevsuren B, Batsuren D, Reist M, Carrupt P-A, Hostettmann K (2008). Xanthones from Gentianella amarella ssp.acuta with acetylcholinesterase and monoamine oxidase inhibitory activities. J. Nat. Prod..

[63] Cho JK, Ryu YB, Curtis-Long MJ, Ryu HW, Yuk HJ, Kim DW, Kim HJ, Lee WS, Park KH (2012). Cholinesterase inhibitory effects of geranylated flavonoids from Paulownia tormentosa fruits. Bioorg. Med. Chem..

[64] Jung HA, Jin SE, Park JS, Choi JS (2011). Antidiabetic complications and anti-alzheimer activities of sophoflavescenol, a prenylated flavonol from Sophora flavescens, and its structure-activity relationship. Phytother. Res..

[65] Kim JY, Lee WS, Kim YS, Curtis-Long MJ, Lee BW, Ryu YB, Park KH (2011). Isolation of cholinesterase-inhibiting flavonoids from Morus lhou. J. Agric. Food Chem..

[66] Ryu HW, Curtis-Long MJ, Jung S, Jeong IY, Kim DS, Kang KY, Park KH (2012). Anticholinesterase potential of flavonols from paper mulberry (Broussonetia papyrifera) and their kinetic studies. Food Chem..

[67] Conforti F, Rigano D, Menichini F, Loizzo MR, Senatore F (2009). Protection against neurodegenerative diseases of Iris pseudopumila extracts and their constituents. Fitoterapia.

[68] Ahmad VU, Iqbal S, Nawaz SA, Choudhary MI, Farooq U, Ali ST, Ahmad A, Bader S, Kousar F, Arshad S, Tareen RB (2006). Isolation of four new pterocarpans from Zygophyllum eurypterum (syn.Z. atriplicoides) with enzyme-inhibition properties. Chem. Biodivers..

[69] Changwong N, Sabphon C, Ingkaninan K, Sawasdee P (2012). Acetyl- and butyryl-cholinesterase inhibitory activities of Mansorins and Mansonones. Phytother. Res..

[70] Nag G, De B (2011). Acetylcholinesterase inhibitory activity of Terminalia chebula, Terminalia bellerica and Emblica officinalis and some phenolic compounds. Int. J. Pharm. Pharm. Sci..

[71] Ge HM, Zhu CH, Shi DH, Zhang LD, Xie DQ, Yang J, Ng SW, Tan RX (2008). Hopeahainol A.An acetylcholinesterase inhibitor from Hopea hainanensis. Chemistry.

[72] Sancheti S, Um BH, Seo SH (2010). 1,2,3,4,6-penta-O-galloyl-ß-D-glucose A cholinesterase inhibitor from Terminalia chebula. S. Afr. J. Bot..

[73] Akhtar MN, Lam KW, Abas F, Maulidiani H, Ahmad S, Shah SA, Rahman AU, Choudhary MI, Lajis NH (2011). New class of acetylcholinesterase inhibitors from the stem bark of Knema laurina and their structural insights. Bioorg. Med. Chem. Lett..

[74] Adhami H R, Linder T, Kaehlig H, Schuster D, Zehl M, Krenn L (2012). Cathechol alkenyls from Semecarpus anacardium.Acetylcholinesterase inhibition and binding mode predictions.. J. Ethnopharmacol.

[75] Jung HA, Jung YJ, Hyun SK, Min BS, Kim DW, Jung JH, Choi JS (2010). Selective cholinesterase inhibitory activities of a new monoterpene diglycoside and other constituents from Nelumbo nucifera stamens. Biol. Pharm. Bull..

[76] Adewusi EA, Moodley N, Steenkamp V (2010). Medicinal plants with cholinesterase inhibitory activity a review. Afr. J. Biotechnol..

[77] BarbosaFilho JM, Medeiros KCP, Diniz MF, Batista LM, Athayde-Filho PF, Silva MS, da-Cunha EVL, Silva Almeida JRG, Quintans-Júnior LJ (2006). Natural products inhibitors of the enzyme acetylcholinesterase. Braz. J. Pharmacogn..

[78] Mukherjee PK, Kumar V, Houghton PJ (2007). Screening of Indian medicinal plants for acetylcholinesterase inhibitory activity. Phytother. Res..

[79] Tundis R, Menichini F, Conforti F, Loizzo MR, Bonesi M, Statti G, Menichini F (2009). A potential role of alkaloid extracts from Salsola species (Chenopodiaceae) in the treatment of Alzheimer's disease. J. Enzyme Inhib. Med. Chem..

[80] Elufioye TO, Obuotor EM, Sennuga AT, Agbedahunsi JM, Adesanya SA (2010). Acetylcholinesterase and butyrylcholinesterase inhibitory activity of some selected Nigerian medicinal plants. Rev. Bras. Farmacogn. . DOI: 10.1590/S0102-695X2010000400002.

[81] Fawole OA, Amoo SO, Ndhlala AR, Light ME, Finnie JF, Van Staden J (2010 ). Anti-inflammatory, anticholinesterase, antioxidant and phytochemical properties of medicinal plants used for pain-related ailments in South Africa. J. Ethnopharmacol..

[82] Cahlíková L, Benešová N, Macáková K, Urbanová K, Opletal L (2011). GC/MS analysis of three Amaryllidaceae species and their cholinesterase activity. Nat. Prod. Commun..

[83] Cahlíková L, Valterová I, Macáková K, Opletal L (2011). Analysis of Amaryllidaceae alkaloids from Zephyranthes grandiflora by GC/MS and their cholinesterase activity. Rev. Bras. Farmacogn. . DOI: 10.1590/S0102-695X2011005000089.

[84] Moyo M, Ndhlala AR, Finnie JF, VanStaden J (2010). Phenolic composition, antioxidant and acetylcholinesterase inhibitory activities of Sclerocarya birrea and Harpephyllum caffrum (Anacardiaceae) extracts. Food Chem..

[85] Benamar H, Rached W, Derdour A, Marouf A (2010). Screening of Algerian medicinal plants for acetylcholinesterase inhibitory activity. J. Biol. Sci..

[86] Lima JA, Costa RS, Epifânio RA, Castro NG, Rocha MS, Pinto AC (2009). Geissospermum vellosii stembark.Anticholinesterase activity and improvement of scopolamine-induced memory deficits. Pharmacol. Biochem. Be..

[87] Yang Z, Zhang D, Ren J, Yang M, Li S (2012). Acetylcholinesterase inhibitory activity of the total alkaloid from traditional Chinese herbal medicine for treating Alzheimer's disease. Med. Chem. Res..

[88] Sekeroglu N, Senol FS, Orhan IE, Gulpinar AR, Kartal M, Sener B (2012). In vitro prospective effects of various traditional herbal coffees consumed in Anatolia linked to neurodegeneration. Food Res. Int..

[89] Loizzo MR (2011,). Radical scavenging activity and cholinesterase inhibitory activity of Leopoldia comosa (L.) bulbs. Progr. Nutr.

[90] Carpinella MC, Andrione DG, Ruiz G, Palacios SM (2010). Screening for acetylcholinesterase inhibitory activity in plant extracts from Argentina. Phytother. Res..

[91] Wszelaki N, Kuciun A, Kiss AK (2010). Screening of traditional European herbal medicines for acetylcholinesterase and butyrylcholinesterase inhibitory activity. Acta Pharm..

[92] Niño J, Hernández JA, Correa YM, Mosquera OM (2006). In vitro inhibition of acetylcholinesterase by crude plant extracts from Colombian flora. Mem. Inst. Oswaldo Cruz..

[93] Bakthir H, Ali NAA, Arnold N, Teichert A, Wessjohann L (2011). Anticholinesterase activity of endemic plant extracts from Soqotra. Afr. J. Tradit. Complement. Altern. Med..

[94] Habtemariam S (2011). The therapeutic potential of Berberis darwinii stem-bark: Quantification of berberine and in vitro evidence for Alzheimer's disease therapy. Nat. Prod. Commun..

[95] Ashraf M, Ahmad K, Ahmad I, Ahmad S, Arshad S, Shah SM, Nasim F (2011). Acetylcholinesterase and NADH oxidase inhibitory activity of some medicinal plants. J. Med. Plant. Res..

[96] Adewusi EA, Moodley N, Steenkamp V (2011). Antioxidant and acetylcholinesterase inhibitory activity of selected southern African medicinal plants. S. Afr. J. Bot..

[97] Akkol EK, Orhan IE, Yesilada E (2012). Anticholinesterase and antioxidant effects of the ethanol extract, ethanol fractions and isolated flavonoids from Cistus laurifolius L. leaves. Food Chem..

[98] Feitosa CM, Freitas RM, Luz NNN, Bezerra MZB, Trevisan MTS (2011). Acetylcholinesterase inhibition by some promising Brazilian medicinal plants. Braz. J. Biol..

[99] Tavares L, McDougall GJ, Fortalezas S, Stewart D, Ferreira RB, Santos CN (2012). The neuroprotective potential of phenolic-enriched fractions from four Juniperus species found in Portugal. Food Chem..

[100] Lee S-H, Sancheti SA, Bafna MR, Sancheti SS, Seo S-Y (2011). Acetylcholineterase inhibitory and antioxidant properties of Rhododendron yedoense var.poukhanense bark. J. Med. Plants Res..

[101] Kwon S-H, Lee H-K, Kim JA, Hong SI, Kim S-Y, Jo T-H, Park Y-I, Lee C-K, Kim Y-B, Lee S-Y, Jang C-G (2011). Neuroprotective effects of Eucommia ulmoides Oliv.bark on amyloid beta25 35-induced learning and memory impairments in mice. Neurosci. Lett..

[102] Crowch CM, Okello EJ (2009). Kinetics of acetylcholinesterase inhibitory activities by aqueous extracts of Acacia nilotica (L. and Rhamnus prinoides (L H r.). Afr. J. Pharm. Pharmacol..

[103] Kim DH, Yoon BH, Kim YW, Lee S, Shin BY, Jung JW, Kim HJ, Lee YS, Choi JS, Kim SY, Lee KT, Ryu JH (2007). The seed extract of Cassia obtusifolia ameliorates learning and memory impairments induced by scopolamine or transient cerebral hypoperfusion in mice. J. Pharmacol. Sci..

[104] Lin HQ, Ho MT, Lau LS, Wong KK, Shaw PC, Wan DC (2008). Anti-acetylcholinesterase activities of traditional Chinese medicine for treating Alzheimer’s disease. Chem. Biol. Interact..

[105] Rauter AP, Martins A, Lopes R, Ferreira J, Serralheiro LM, Araújo ME, Borges C, Justino J, Silva FV, Goulart M, Thomas-Oates J, Rodrigues JA, Edwards E, Noronha JP, Pinto R, Mota-Filipe H (2009). Bioactivity studies and chemical profile of the antidiabetic plant Genista tenera. J. Ethnopharmacol..

[106] Satheeshkumar N, Mukherjee PK, Bhadra S, Saha BP (2010). Acetylcholinesterase enzyme inhibitory potential of standardized extract of Trigonella foenum graecum L and its constituents. Phytomedicine.

[107] Bhadra S, Mukherjee PK, Kumar NS, Bandyopadhyay A (2011). Anticholinesterase activity of standardized extract of Illicium verum Hook.f. fruits. Fitoterapia.

[108] Orhan I, Senol FS, Gülpinar AR, Kartal M, Sekeroglu N, Deveci M, Kan Y, Sener B (2009). Acetylcholinesterase inhibitory and antioxidant properties of Cyclotrichium niveum, Thymus praecox subsp.caucasicus var. caucaicusEchinacea purpurea and E. pallida. Food Chem. Toxicol.

[109] Costa P ( 2011). Inhibitory effect of Lavandula viridis on Fe2+-induced lipid peroxidation, antioxidant and anti-cholinesterase properties. Food Chem.

[110] Orhan IE, Belhattab R, Senol FS, Gülpinar AR, Hosbas S ( 2010). Profiling of cholinesterase inhibitory and antioxidant activities of Artemisia absinhiumA. herba-alba, A. fragrans, Marrubium vulgare, M. astranicum, Origanum vulgare subsp. glandulossum and essential oil analysis of two Artemisia species. Ind. Crop. Prod..

[111] Loizzo MR ( 2009). Menichini, F.Chemical analysis, antioxidant, antiinflammatory and anticholinesterase activities of Origanum ehrenbergii Boiss and Origanum syriacum L. essential oils. Food Chem.

[112] Mossa AT, Nawwar GA (2011). Free radical scavenging and antiacetylcholinesterase activities of Origanum majorana L. essential oil. Hum. Exp. Toxicol..

[113] Tel G, Oztürk M, Duru ME, Harmandar M, Topçu G (2010). Chemical composition of the essential oil and hexane extract of Salvia chionantha and their antioxidant and anticholinesterase activities. Food Chem. Toxicol..

[114] Senol FS ( 2010). Survey of 55 Turkish Salvia taxa for their acetylcholinesterase inhibitory and antioxidant activities. Food Chem.

[115] Loizzo MR, Menichini F, Tundis R, Bonesi M, Conforti F, Nadjafi F, Statti GA, Frega NG, Menichini F (2009). In vitro biological activity of Salvia leriifolia benth essential oil relevant to the treatment of Alzheimer's disease. J. Oleo Sci..

[116] Ahmad B, Mukarram Shah SM, Khan H, Hassan Shah SM (2007). Enzyme inhibition activities of Teucrium royleanum. J. Enzym. Inhib. Med. Ch..

[117] Tappayuthpijarn P, Itharat A, Makchuchit S (2011). Acetylcholinesterase inhibitory activity of Thai traditional nootropic remedy and its herbal ingredients. J. Med. Assoc. Thai..

[118] Vinutha B, Prashanth D, Salma K, Sreeja SL, Pratiti D, Padmaja R, Radhika S, Amit A, Venkateshwarlu K, Deepak M (2007). Screening of selected Indian medicinal plants for acetylcholinesterase inhibitory activity. J. Ethnopharmacol..

[119] Ashraf M, Ahmad K, Ahmad I, Ahmad S, Arshad S, Shah SM, Nasim F (2011). Acetylcholinesterase and NADH oxidase inhibitory activity of some medicinal plants. J. Med. Plant. Res..

[120] Adsersen A, Gauguin B, Gudiksen L, Jäger AK (2006). Screening of plants used in Danish folk medicine to treat memory dysfunction for acetylcholinesterase inhibitory activity. J. Ethnopharmacol..

[121] Ustun O, Senol F, Kurkcuoglu M, Orhan I, Kartal M, Baser K (2012). Investigation on chemical composition, anticholinesterase and antioxidant activities of extracts and essential oils of Turkish Pinus species and pycnogenol. Ind. Crop. Prod..

[122] Bonesi M, Menichini F, Tundis R, Loizzo MR, Conforti F, Passalacqua NG, Statti GA, Menichini F (2010). Acetylcholinesterase and butyrylcholinesterase inhibitory activity of Pinus species essential oils and their constituents. J. Enzym. Inhib. Med. Ch..

[123] Khadri A, Serralheiro MLM, Nogueira JMF, Neffati M, Smiti S, Araújo MEM (2008). Antioxidant and antiacetylcholinesterase activities of essential oils from Cymbopogon schoenanthus L.Spreng. Determination of chemical composition by GC mass spectrometry and 13C NMR. Food Chem..

[124] Khadri A, Neffati M (2010). Antioxidant, antiacetylcholinesterase and antimicrobial activities of Cymbopogon schoenanthus L. Spreng (lemon grass) from Tunisia. LWT-Food Sci. Technol..

[125] Aremu AO, Amoo SO, Ndhlala AR, Finnie JF, Stadenm JV (2011). Antioxidant activity, acetylcholinesterase inhibition, iridoid content and mutagenic evaluation of Leucosidea sericea. Food Chem. Toxicol..

[126] Pachauri SD, Tota S, Khandelwal K, Verma PR, Nath C, Hanif K, Shukla R, Saxena JK, Dwivedi AK ( 2012). Protective effect of fruits of Morinda citrifolia L.on scopolamine induced memory impairment in mice. A behavoralbiochemical and cerebral blood flow study. J. Ethnopharmacol.

[127] Chaiyana W, Okonogi S (2012). Inhibition of cholinesterase by essential oil from food plant. Phytomedicine.

[128] Menichini F, Tundis R, Bonesi M, de Cindio B, Loizzo MR, Conforti F, Statti GA, Menabeni R, Bettini R, Menichini F ( 2011). Chemical composition and bioactivity of Citrus medica L.cv. Diamante essential oil obtained by hydrodistilltioncold-pressing and supercritical carbon dioxide extraction. Nat. Prod. Res.

